# Evodiae Fructus extract suppresses inflammatory response in HaCaT cells and improves house dust mite-induced atopic dermatitis in NC/Nga mice

**DOI:** 10.1038/s41598-023-50257-3

**Published:** 2024-01-04

**Authors:** Seong Eun Jin, Chang-Seob Seo, Woo-Young Jeon, Yong Jin Oh, Hyeun-Kyoo Shin, Hye Gwang Jeong, Hyekyung Ha

**Affiliations:** 1 KM Science Research Division, Korea Institute of Oriental Medicine, 1672 Yuseong-daero, Yuseong-gu, Daejeon, 34054 Republic of Korea; 2 College of Pharmacy, Chungnam National University, 99 Daehak-ro, Yuseong-gu, Daejeon, 34134 Republic of Korea; 3 KM Convergence Research Division, Korea Institute of Oriental Medicine, 1672 Yuseong-daero, Yuseong-gu, Daejeon, 34054 Republic of Korea

**Keywords:** Immunology, Molecular biology

## Abstract

This study was conducted to assess the effect of Evodiae Fructus 70% ethanol extract (EFE) on the pathology of atopic dermatitis using in vitro and in vivo models. The major compounds in EFE were identified by ultra-performance liquid chromatography with tandem mass spectrometry as rutaecarpine, evodiamine, evodol, dehydroevodiamine, limonin, synephrine, evocarpine, dihydroevocarpine, and hydroxyevodiamine. EFE significantly decreased chemokine levels in tumor necrosis factor-α/interferon-γ-stimulated HaCaT cells. In house dust mite-treated NC/Nga mice, topical application of EFE significantly decreased the dermatitis score, epidermal hyperplasia and thickening, mast cell infiltration, and plasma levels of histamine and corticosterone. Thymic stromal lymphopoietin, CD4^+^ T cells, interleukin-4, and intercellular adhesion molecule-1 expression in the lesioned skin was reduced in the treated mice. The mechanism of EFE was elucidated using transcriptome analysis, followed by experimental validation using Western blotting in HaCaT cells. EFE down-regulated the activation of Janus kinase (JAK)-signal transducers and activators of transcription (STAT) and mitogen-activated protein kinases (MAPK) signaling pathways in HaCaT cells. EFE improves atopic dermatitis-like symptoms by suppressing inflammatory mediators, cytokines, and chemokines by regulating the JAK-STAT and MAPK signaling pathways, suggesting its use as a potential agent for the treatment of atopic dermatitis.

## Introduction

Atopic dermatitis (AD) is a persistent and chronic inflammatory skin disease accompanied by eczematous skin lesions, severe pruritus, and dry skin^1^. The pathogenesis of AD is linked to a complicated interplay between environmental and genetic variables, severe pruritus, skin barrier abnormalities, and immune dysregulation. AD causes intense itchiness, and scratching of the skin causes inflammation, which intensifies itching and worsens clinical symptoms^2^. Patients with AD experience discomfort in their daily lives due to insufficient sleep caused by intense itching, problems in social relationships, and economic issues due to the cost of therapy^3^. The goal of AD treatment is to reduce pruritus and implement persistent disease management that enables patients to be completely functional at home and in society^4^.

During AD development, the immune response is triggered in response to infections, and the body exhibits disease symptoms^5^. T lymphocytes, which mainly infiltrate AD lesions, are classified into Th1 and Th2 cells based on their function. In acute AD, Th2 cells are abundant, and Th2 cytokines such as interleukin (IL)-4, -5, and -31 trigger allergic reactions and suppress innate immune responses^6^. In particular, IL-4 plays a substantial role in the differentiation of naïve T cells into Th2 cells and stimulates B lymphocytes to generate immunoglobulin E (IgE)^7^. IgE activates mast cells by signaling through FcεRI, a high-affinity IgE receptor^7,8^. Mast cells are distributed in relatively large numbers near externally exposed surfaces, including the skin, and are among the first immune cells to react to allergens or other substances. Activated mast cells release a wide range of mediators, including histamine and cytokines, which promote the maturation, functional activation, and migration of dendritic cells, thereby promoting the development of sensitization and influencing the inflammatory response^7–9^. Keratinocytes secrete proinflammatory cytokines and chemokines that allow immune cells to reach inflammatory regions. Therefore, because multiple factors are considered in the pathogenesis of AD, a multi-targeted treatment strategy is required^5^.

Current AD treatment strategies include topical corticosteroids, calcineurin inhibitors, oral steroids, and immunosuppressive agents and focus on alleviating the symptoms of acute inflammation but are usually accompanied by side effects such as skin atrophy and drug resistance development^10^. As the need for safe and effective AD therapies increases, traditional complementary medicines with multiple targets can provide various benefits, such as fewer side effects, reduced costs, and improved patient compliance^5,11^.

Evodiae Fructus, known as the unripe fruit of *Evodia rutaecarpa* Bentham, has been used to treat abdominal pain, vomiting, diarrhea, headache, stomachaches, and dysmenorrhea^12^. Several studies have revealed that Evodiae Fructus has anti-allergic^13^, anti-inflammatory^14^, and antinociceptive effects^15^. According to biochemical analysis studies, Evodiae Fructus includes alkaloids (rutaecarpine and evodiamine), terpenoids (limonin), and flavonoids^16,17^. Numerous pharmacological studies have reported that the components of Evodiae Fructus, such as rutaecarpine, evodiamine, dehydroevodiamine, dihydroevocarpine, and hydroxyevodiamine, possess anti-inflammatory, anti-tumor, anti-atherosclerosis, neuroprotective, and antidepressant properties^17^. Based on previous studies, we hypothesized that Evodiae Fructus could have potential effects on the treatment of inflammatory diseases and reduce the risk of AD.

In the present study, we investigated the anti-inflammatory properties of Evodiae Fructus 70% ethanol extract (EFE) using activated keratinocytes (HaCaT) and its influences on AD-like skin lesions using NC/Nga mice with house dust mite (HDM)-induced AD.

## Materials and methods

### Plant materials and preparation of EFE

Dried Evodiae Fructus originating from China was purchased from Kwangmyeongdang (Ulsan, Korea), a specialized herbal medicine supplier. The raw material was morphologically identified by Dr. Goya Choi of the Korea Institute of Oriental Medicine (Naju, Korea). To prepare the 70% ethanol extract, 2.0 L of 70% ethanol was added to 1.0 kg of dried Evodiae Fructus, followed by ultrasonic extraction at room temperature for 1 h. The extract was filtered through a standard sieve (270 mesh) and the solvent was removed from the filtered solution using a rotary evaporator (SciLab Korea Co., Ltd., EV-1020, Wonju, Korea). The residue was suspended in distilled water and lyophilized using a freeze dryer (ilShinBioBase Co., Ltd., PVTFD-100, Yangju, Korea) to obtain a powdered sample weighing 183.17 g (yield: 18.32%; lot number: K0282210330; specimen number: EBM157). Our study including the plant materials complies with institutional, national, and international guidelines and legislation.

### Chemicals and reagents

Information on the chemicals and reagents used for in vitro and in vivo experiments is presented in the Supplementary Materials (Supplementary Tables S1 and S2).

### UPLC-MS/MS simultaneous analysis of compounds in EFE

The sample solution for the simultaneous analysis of nine compounds (rutaecarpine, evodiamine, evodol, dehydroevodiamine, limonin, synephrine, evocarpine, dihydroevocarpine, and hydroxyevodiamine; Supplementary Fig. S1) in EFE using ultra-performance liquid chromatography with tandem mass spectrometry (UPLC-MS/MS) was prepared by adding 10 mL of 70% methanol to 100 mg of the lyophilized sample, followed by ultrasonic extraction for 5 min and vortexing for 1 min. A standard solution of each of the nine compounds was prepared at a concentration of 100 μg/mL using methanol and stored in a refrigerator (4 °C). All solutions were filtered through a hydrophobic polytetrafluoroethylene syringe filter (pore: 0.22 μm; housing diameter: 13 mm; SSOLKorea, Daejeon, Korea) prior to analysis.

Simultaneous quantitation of the nine compounds in EFE was performed using a UPLC-MS/MS system consisting of an Acquity UPLC system and an Xevo TQ-XS mass spectrometry (MS) system (Waters, Milford, MA, USA). Marker compounds were separated on a Waters Acquity UPLC BEH C_18_ column (2.1 mm × 100 mm, 1.7 μm, Milford, MA, USA) maintained at 45 °C using a mobile phase of a 0.1% (v/v) aqueous formic acid with 5 mM ammonium formate– acetonitrile system. Each compound was detected in multiple reaction monitoring (MRM) mode using an electrospray ionization (ESI) source with a Xevo TT-XS MS system. The detailed UPLC and MS analysis conditions for the simultaneous analysis are shown in Supplementary Tables S3, S4, and the UPLC-MS/MS MRM parameters are presented in Supplementary Table S5. Data acquisition and processing were controlled using MassLynx software (Version 4.2, Milford, MA, USA).

### Measurement of chemokine levels in HaCaT cells

The human keratinocyte cell line (HaCaT) was obtained from the CLS Cell Lines Service GmbH (Eppelheim, Baden-Württemberg, Germany) and cultured as described previously^18^. Cell viability was measured using the Cell Counting Kit-8 (CCK-8) assay, and the detailed methods are presented in the Supplementary Materials (Supplementary Fig. S2). To analyze the effects of EFE and its compounds on the release of chemokines, the cells were incubated in 6-well plates (1 × 10^6^ cells/well) with various concentrations of the EFE and its compounds in the presence of tumor necrosis factor (TNF)-α and interferon (IFN)-γ (TI, each 10 ng/mL) for 24 h. The levels of regulated on activation, normal T cell expressed, and secreted (RANTES; CCL5), thymus- and activation-regulated chemokines (TARC; CCL17), and macrophage-derived chemokines (MDC; CCL22) in the culture supernatants were determined using enzyme-linked immunosorbent assay (ELISA) kits. Silymarin was used as a positive control according to a previous study^19^. Data were analyzed using one-way analysis of variance (ANOVA) followed by a post-hoc Bonferroni test using SYSTAT software (Version 13.1, SYSTAT Software, Inc., San Jose, CA, USA). Differences were considered statistically significant at *p* values less than 0.05.

### Experiment on the influence of EFE in HDM-induced AD mouse model

#### Animals

Specific pathogen-free NC/Nga mice (male, nine-weeks-old) were obtained from Central Laboratory Animals Inc. (Seoul, Korea) and acclimatized for a week prior to the start of the experiment. Animals were maintained in a temperature-controlled room at 23 ± 3 °C with a relative humidity of 40–60% on a 12 h light/dark cycle. Water and commercial rodent chow were provided ad libitum.

#### Induction of atopic dermatitis and drug treatment

The mice were divided into six groups as follows: normal control (NC; n = 7), AD (n = 8), prednisolone (positive control, PC; 0.2 mg/mouse; n = 7), and EFE (1 and 3 mg/mouse; n = 7/group). A schematic representation of the experimental schedule is shown in Supplementary Fig. S3. Prior to the experiment, the backs of the mice were shaved. To induce AD-like skin lesions, HDM extract (*Dermatophagoides farinae* extract, Biostir-AD^®^)^20^ was applied to the dorsal skin and both surfaces of each ear of all mice, except the NC group, twice per week for four weeks, as previously reported^21^. Prednisolone, a corticosteroid, was used as a positive control group. For four weeks, prednisolone and EFE dissolved in 70% ethanol were applied topically once daily. NC and AD groups were topically treated with 70% ethanol. The volume of 70% ethanol solution applied topically in each group was 150 μL per mouse. Eighteen hours after the last application, the mice were anesthetized by intraperitoneal injection of pentobarbital sodium (Entobar; Hanlim Pharm. Co., Ltd., Seoul, Republic of Korea), and blood and tissue were collected.

#### Evaluation of dermatitis severity, body weight, and the spleen index

Every week, body weight was measured and dermatitis severity was assessed macroscopically in a blinded study according to the Eczema Area and Severity Index (EASI) scoring system^22^. The final score was determined by summarizing the individual scores for the symptoms of erythema/hemorrhage, scarring/dryness, edema, and excoriation/erosion. The spleen index was calculated as the ratio of spleen to body weight.

#### Measurement of plasma histamine, IgE, and corticosterone levels

Plasma levels of histamine, total IgE, and corticosterone were measured using ELISA kits following the manufacturer’s instructions.

#### Histological and immunohistochemical analysis

Histological and immunohistochemical analyses were performed as previously described^21^. Briefly, the dorsal skin and ear were fixed in 10% neutral-buffered formalin for histological and immunohistochemical (IHC) analyses. Paraffin-embedded tissues were sliced into 4 μm-thick sections. The sections were stained with hematoxylin and eosin (H&E) to investigate histological features. The epidermal thickness of the dorsal skin and ear was measured in three randomly selected areas on the H&E-stained slides using the Motic VM 3.0-Motic Digital Slide Assistant (Version 1.0.7.60, Motic China Group Co., Ltd.) with Motic EasyScan (Motic, Hong Kong). To assess mast cell infiltration, dorsal skin and ear sections were stained with toluidine blue (TB). The number of purple-stained mast cells on the slides stained with TB was counted in two randomly selected areas.

Dorsal skin sections were immunostained with antibodies against thymic stromal lymphopoietin (TSLP), CD4^+^ T cells, IL-4, and intercellular adhesion molecule-1 (ICAM-1) antibodies. All stained slides were visualized using Motic EasyScan (Motic Asia, Hong Kong) and IHC analysis was performed using the Motic Digital Slide Assistant (Motic VM 3.0, Version 1.0.7.60, Motic China Group Co., Ltd.). Dark brown areas indicate positively stained cells. Quantitative morphometric analysis of the immunostained regions in relation to the total area was performed (MetaMorph Offline, Version 7.7.0.0, Molecular Devices, Inc.). Statistical analysis was performed in the same manner as in “Measurement of chemokine levels in HaCaT cells” section.

### Transcriptomic analysis in HaCaT cells

#### RNA-sequencing (RNA-Seq) and differentially expressed genes (DEGs) analysis

As described in “Measurement of chemokine levels in HaCaT cells” section, chemokine was measured using culture supernatants, and transcriptome analysis was performed using cells. After collecting supernatants, cells were washed with ice-cold phosphate buffered saline and sent to DNA Link Inc. (Seoul, Korea) for extraction of total RNA and determination of RNA quality. RNA-sequencing (RNA-Seq) was analyzed at KaiPharm Co., Ltd. (Seoul, Korea). Raw-fastq were trimmed using fastp (Version 0.21.0) with Illumina TruSeq Library adapter sequences. Trimmed and low-quality reads with eliminated fastq were aligned with the reference genome using STAR aligner (Version 2.7.1a) by two-pass mode. The Ensembl (Version 96) genome was used as a reference for a customized gene transfer format annotation file. Thereafter, gene counts, transcripts per million mapped reads, and fragments per kilobase of transcript per million mapped reads were determined using high-throughput sequence (HTSeq; Version 0.12.3) and Salmon (Version 1.2.1). Counts per million mapped reads and DEGs were calculated using R packages edgeR and DESeq2, respectively (logFC > 1.3 and *p* value < 0.01). Before running DESeq2, low expressed genes were filtered out using edgeR, and DEGs were assigned using in-house scoring method based on the results of DESeq2 analysis.

#### Pathway analysis

Customize pathway datasets were manually curated using public databases, including the Kyoto Encyclopedia of Genes and Genomes (KEGG), Gene Ontology (GO) biological process, Molecular Signature Database (MSigDB), Chemical Genetic Perturbation, Oncogenic Signature, EMTome, and Secretome. Thereafter, the datasets were analyzed using over-representation analysis (ORA) and gene set enrichment analysis (GSEA). In ORA, pathways that were altered by DEGs were identified using in-house scores based on enrichment-factors and hypergeometric *p* values. The significantly enriched gene sets were identified using normalized enrichment score and *p* values combined scores using the fgsea package of R.

#### Experimental validation

To further explore the anti-AD mechanism of action (MoA) of EFE in TI-stimulated HaCaT cells, Western blotting was performed for the Janus kinase (JAK) 1, signal transducers and activators of transcriptions (STATs; STAT1, STAT3, STAT5, and STAT6), and mitogen-activated protein kinases (MAPKs; p38, extracellular signal-regulated kinase (ERK), and c-Jun N-terminal kinase (JNK)). Total cell lysates were prepared and analyzed using western blotting, as previously reported^19^. Data were analyzed by one-way ANOVA, followed by a Fisher’s Least Significant Difference post hoc test using SYSTAT software. Differences were considered statistically significant if *p* values less than 0.05.

### Ethical approval

The animal study was performed in accordance with the NIH Guidelines for the Care and Use of Laboratory Animals^23^ and approved by the Institutional Animal Care and Use Committee of the Korea Institute of Oriental Medicine (approval number: #22-076). This study is reported in accordance with ARRIVE guidelines (https://arriveguidelines.org).

## Results

### Simultaneous analysis of nine marker compounds in EFE by UPLC-MS/MS

**Figure 1:**
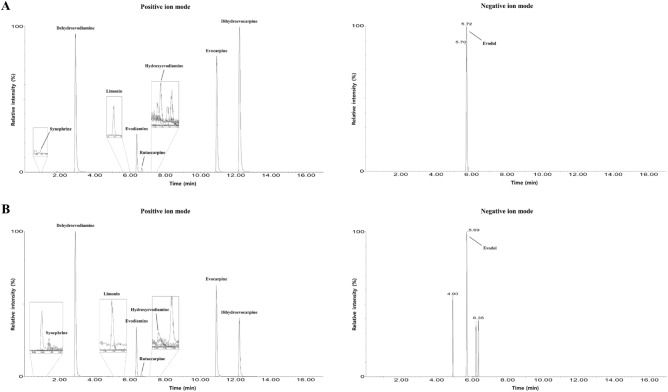
Total ion chromatograms of the mixed standard solutions of nine compounds (**A**) and 70% methanolic solution of lyophilized EFE (**B**) measured by UPLC–MS/MS MRM in positive and negative ion modes. The concentration of each compound in the standard solution was 1.0 μg/mL. Comp.1, rutaecarpine; Comp.2, evodiamine; Comp.3, evodol; Comp.4, dehydroevodiamine; Comp.5, limonin; Comp.6, synephrine; Comp.7, evocarpine; Comp.8, dihydroevocarpine; Comp.9, hydroxyevodiamine; EFE, Evodiae Fructus 70% ethanol extract; MRM, multiple reaction monitoring; UPLC-MS/MS, ultra-performance liquid chromatography with tandem mass spectrometry.

The optimized UPLC-MS/MS MRM assay was suitable for the simultaneous analysis of the nine compounds in EFE, all of which were detected within 12.23 min (Fig. 1). Evodol was detected in the negative ion mode of the [M-H]^–^ form at *m/z* 483.3, and eight compounds (rutaecarpine, evodiamine, dehydroevodiamine, limonin, synephrine, evocarpine, dihydroevocarpine, and hydroxyevodiamine) were detected in the positive ion mode of the [M + H]^+^ form at *m/*z 288.2, 304.3, 302.3, 471.2, 168.1, 340.4, 342.4, and 320.4, respectively (Fig. 1 and Supplementary Table S4). In the regression equation developed for the quantitation of the analyte in the concentration range tested for each analyte, the coefficient of determination (*r*^2^) was ≥ 0.9951, indicating good linearity (Supplementary Table S5). The limits of detection (LOD) and quantitation (LOQ) were calculated as 0.02–3.05 μg/L and 0.05–9.15 μg/L, which were calculated as signal-to-noise ratios of 3 and 10, respectively (Supplementary Table S5). As a result of analyzing nine compounds in EFE by applying the above UPLC-MS/MS MRM assay, nine compounds showed a content of 0.01–8.78 mg/g (Table 1).

**Table 1:** Quantification of compounds in EFE using the UPLC–MS/MS MRM analytical method.

Code No.	Name	Amount (mg/g)	RSD (%)
Comp.1	Rutaecarpine	7.14 ± 0.60	8.36
Comp.2	Evodiamine	8.78 ± 0.49	5.53
Comp.3	Evodol	6.30 ± 0.53	8.48
Comp.4	Dehydroevodiamine	7.71 ± 0.32	4.14
Comp.5	Limonin	3.00 ± 0.20	6.81
Comp.6	Synephrine	0.64 ± 0.05	7.88
Comp.7	Evocarpine	6.45 ± 0.54	8.45
Comp.8	Dihydroevocarpine	2.11 ± 0.19	9.01
Comp.9	Hydroxyevodiamine	0.01 ± 0.00	6.37

Data are expressed as the mean ± standard deviation (n = 3). *EFE* Evodiae Fructus 70% ethanol extract, *MRM* multiple reaction monitoring, *RSD* relative standard deviation, *UPLC-MS/MS* ultra-performance liquid chromatography with tandem mass spectrometry.

### Effect of EFE and its compounds on the production of chemokines in HaCaT cells

**Figure 2:**
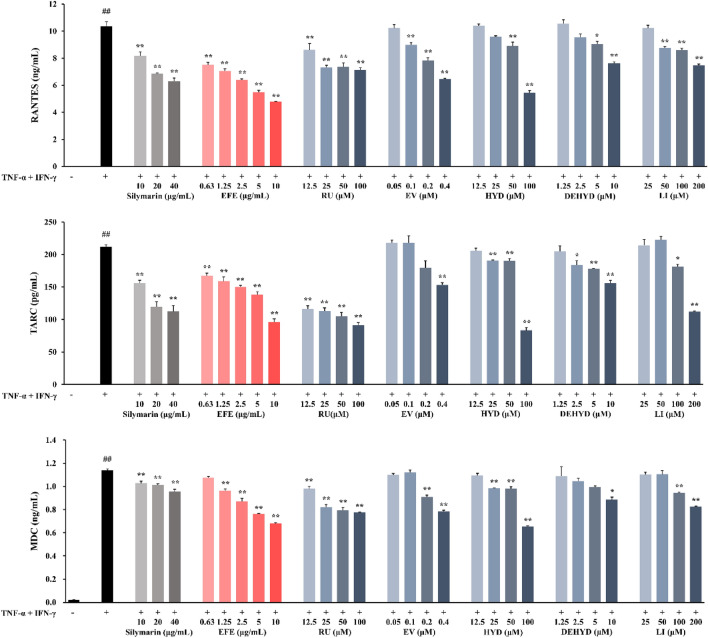
Effect of EFE and its compounds on the production of chemokines in TI-stimulated HaCaT cells. The cells were treated with EFE and its compounds, and stimulated with TI for 24 h. The levels of RANTES (**A**), TARC (**B**), and MDC (**C**) in the supernatant were measured using ELISA kits. Silymarin was used as a positive control. Data are expressed as mean ± SEM (n = 3). ^##^*p* < 0.01 versus NC; *p < 0.05 and **p < 0.01 versus TI-stimulated cells. *DEHYD* dehydroevodiamine, *EFE* Evodiae Fructus 70% ethanol extract, *EV* evodiamine, *HYD* hydroxyevodiamine, *LI* limonin, *NC* normal control, *RU* rutaecarpine, *TI* TNF-α (10 ng/mL) and IFN-γ (10 ng/mL).

To investigate the effects of EFE and its compounds on TI-induced skin inflammation in HaCaT cells, we evaluated the levels of RANTES, TARC, and MDC. All experiments used non-toxic concentrations of EFE and its compounds (Supplementary Fig. S4). The levels of RANTES, TARC, and MDC were increased by TI (*p* < 0.01; Fig. 2). In contrast, silymarin, which was used as a positive control, inhibited this effect (*p* < 0.01). Compared to the TI group, treatment with EFE (IC_50_ values: RANTES, 6.53 μg/mL; TARC, 8.84 μg/mL; MDC, > 10 μg/mL) and its five active compounds included in EFE, namely rutaecarpine, evodiamine, hydroxyevodiamine, dehydroevodiamine, and limonin, significantly suppressed the levels of RANTES, TARC, and MDC in TI-stimulated HaCaT cells (*p* < 0.05; Fig. 2). These five active compounds were chosen based on a preliminary study that confirmed the effects of EFE and its nine ingredients in TI-stimulated HaCaT cells (Supplementary Fig. S5).

### Effects of topically applied EFE on AD-like skin lesions in NC/Nga mice

#### Effects of EFE on dermatitis severity, body weight, and the spleen index

**Figure 3:**
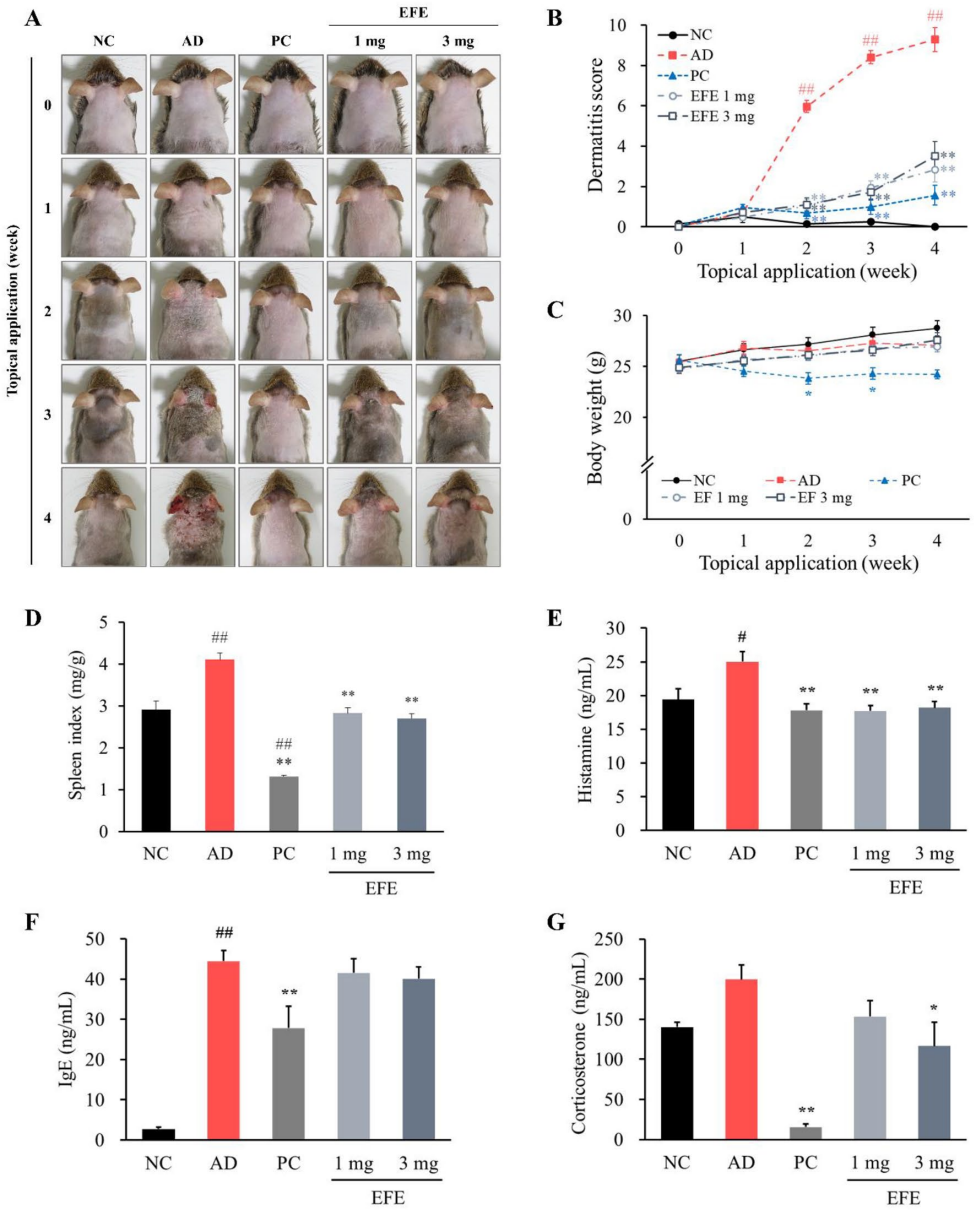
Effects of EFE on skin lesions, spleen index, and plasma biomarkers in HDM-induced AD mice. (**A**) Photographic images of the skin lesions on each mouse. (**B**) Dermatitis scores were evaluated weekly for 4 weeks. Dermatitis scores were calculated as sums of individual scores, graded as 0 (none), 1 (mild), 2 (moderate), and 3 (severe) for each of the four symptoms; erythema/hemorrhage, scarring/dryness, edema, and excoriation/erosion. Changes in body weight (**C**) and the spleen index (**D**) from each group. The levels of histamine (**E**), IgE (**F**), and corticosterone (**G**) in plasma were measured using ELISA kits. Data are expressed as mean ± SEM (n = 7–8). ^#^*p* < 0.05 and ^##^*p* < 0.01, significantly different from the NC; *p < 0.05 and **p < 0.01, significantly different from the AD. *AD* atopic dermatitis, *EFE* Evodiae Fructus 70% ethanol extract, *HDM* house dust mite, *NC* normal control, *PC* positive control.

To investigate the in vivo effect of EFE on AD-like skin inflammation, an HDM-induced AD mouse model was used. Topical application of HDM produced remarkable AD-like lesions, including erythema/hemorrhage, scarring/dryness, edema, and excoriation/erosion. Treatment with prednisolone and EFE (1 and 3 mg) restored skin inflammation and decreased the dermatitis score at weeks 2, 3, and 4 compared to the AD group (*p* < 0.01; Fig. 3A,B). Prednisolone treatment resulted in body weight decrease (*p* < 0.01), but there was not a noticeable difference in body weight in the other groups (Fig. 3C). A comparison of spleen indices to determine the immune system status showed that EFE treatment reduced the high spleen index to normal levels (*p* < 0.01; Fig. 3D). By contrast, treatment with prednisolone resulted in a significantly lower spleen index than in the normal control (*p* < 0.01).

#### Effect of EFE on the level of histamine, IgE, and corticosterone

To investigate whether EFE influences systemic inflammation in an AD model, we evaluated histamine and total IgE levels in plasma. Plasma histamine and total IgE levels were significantly increased by HDM treatment in NC/Nga mice (*p* < 0.05 and *p* < 0.01; Fig. 3E,F). Topical application of prednisolone and EFE (1 and 3 mg) significantly reduced the histamine levels in the plasma compared to the normal control (*p* < 0.01). Total IgE levels were considerably decreased by prednisolone (*p* < 0.01), whereas the EFE at either dose was not statistically different from the AD group.

To determine whether EFE reduced the stress response, we measured the plasma corticosterone levels (Fig. 3G). HDM-induced AD mice showed higher corticosterone levels than normal control mice. When compared with the AD group, corticosterone levels were significantly reduced by treatment with EFE 3 mg (*p* < 0.05). Treatment with prednisolone lowered corticosterone levels (*p* < 0.01) compared to the AD group.

#### Effect of EFE on histopathological features and mast cell infiltration

**Figure 4:**
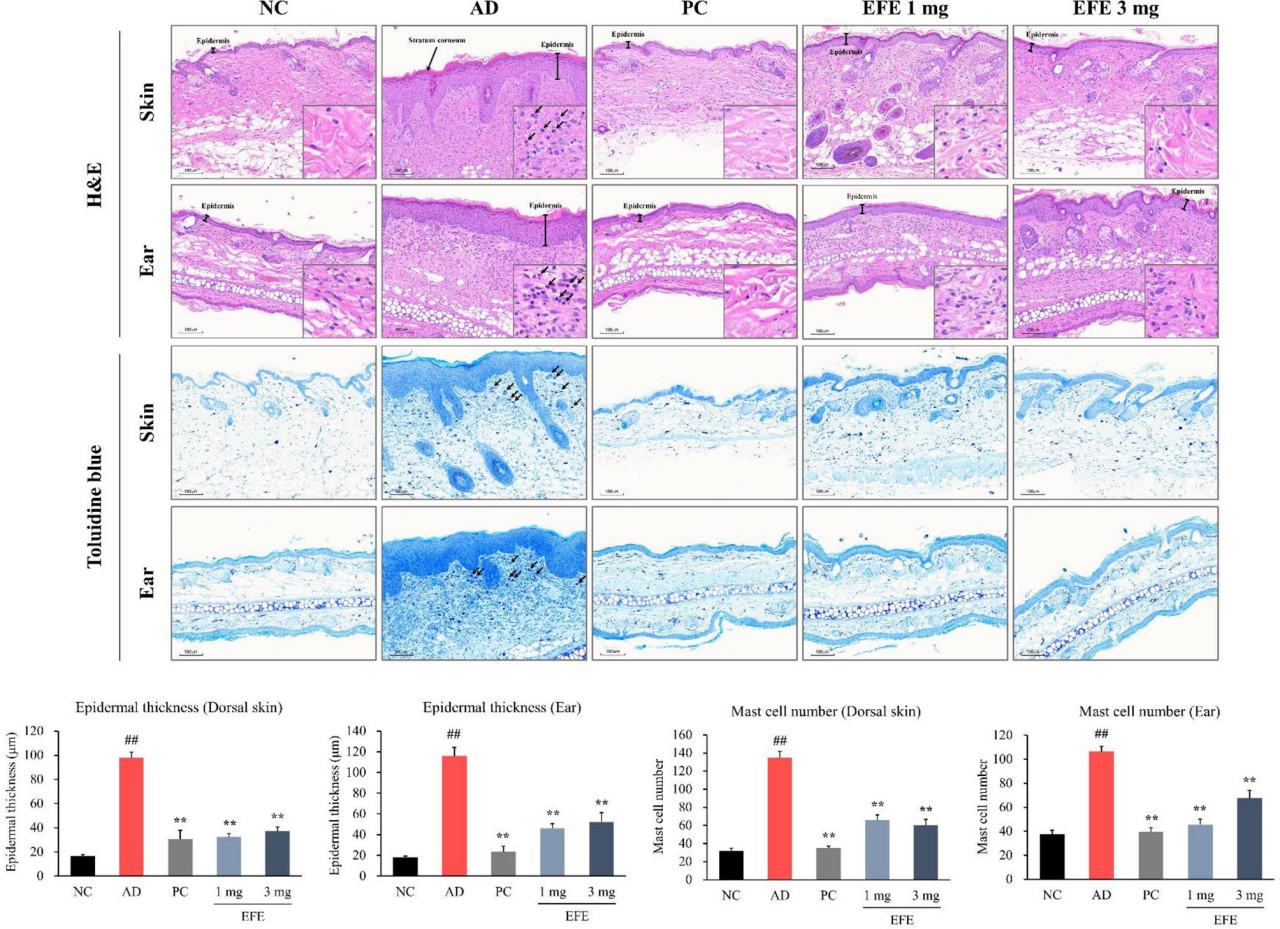
Effect of EFE on the histopathological features and mast cell infiltration of dorsal skin and ear lesions in HDM-induced AD mice. Histological features of lesions were determined with H&E (× 10). Mast cells were stained with toluidine blue (× 10). The epidermal thickness of the dorsal skin and ear was measured in three randomly selected areas on the H&E staining slide. The number of mast cells stained purple was counted in two randomly selected areas on the toluidine blue staining slide of the dorsal skin and ear. Data are presented as mean ± SEM (n = 7–8). ^##^*p* < 0.01, significantly different from the NC; **p < 0.01, significantly different from the AD. Scale bar = 100 μm. *AD* atopic dermatitis, *EFE* Evodiae Fructus 70% ethanol extract, *H&E* hematoxylin and eosin, *HDM* house dust mite, *NC* normal control, *PC* positive control.

The effect of EFE on histological alterations and mast cell infiltration of the lesioned skin was assessed using H&E and TB staining of the dorsal skin and ear. The dorsal skin and ear of the AD group showed inflammatory cell infiltration and aberrant epidermal thickness which were caused by epidermal hyperplasia and keratinization, including hyperkeratosis, compared with the NC group (Fig. 4). Topical prednisolone and EFE 1 and 3 mg treatments greatly improved HDM-induced histological alterations and significantly reduced dorsal skin and ear thickness at week-4 compared to the AD group (*p* < 0.01; Fig. 4).

TB staining showed that mast cell infiltration was significantly increased in the skin lesions of the AD group (*p* < 0.01, Fig. 4). Topical application of prednisolone and EFE (1 and 3 mg) markedly reduced HDM-induced mast cell hyperplasia in the lesional skin (*p* < 0.01).

#### Effect of TSLP, CD4^+^ T cells, IL-4, and ICAM-1 expression

**Figure 5:**
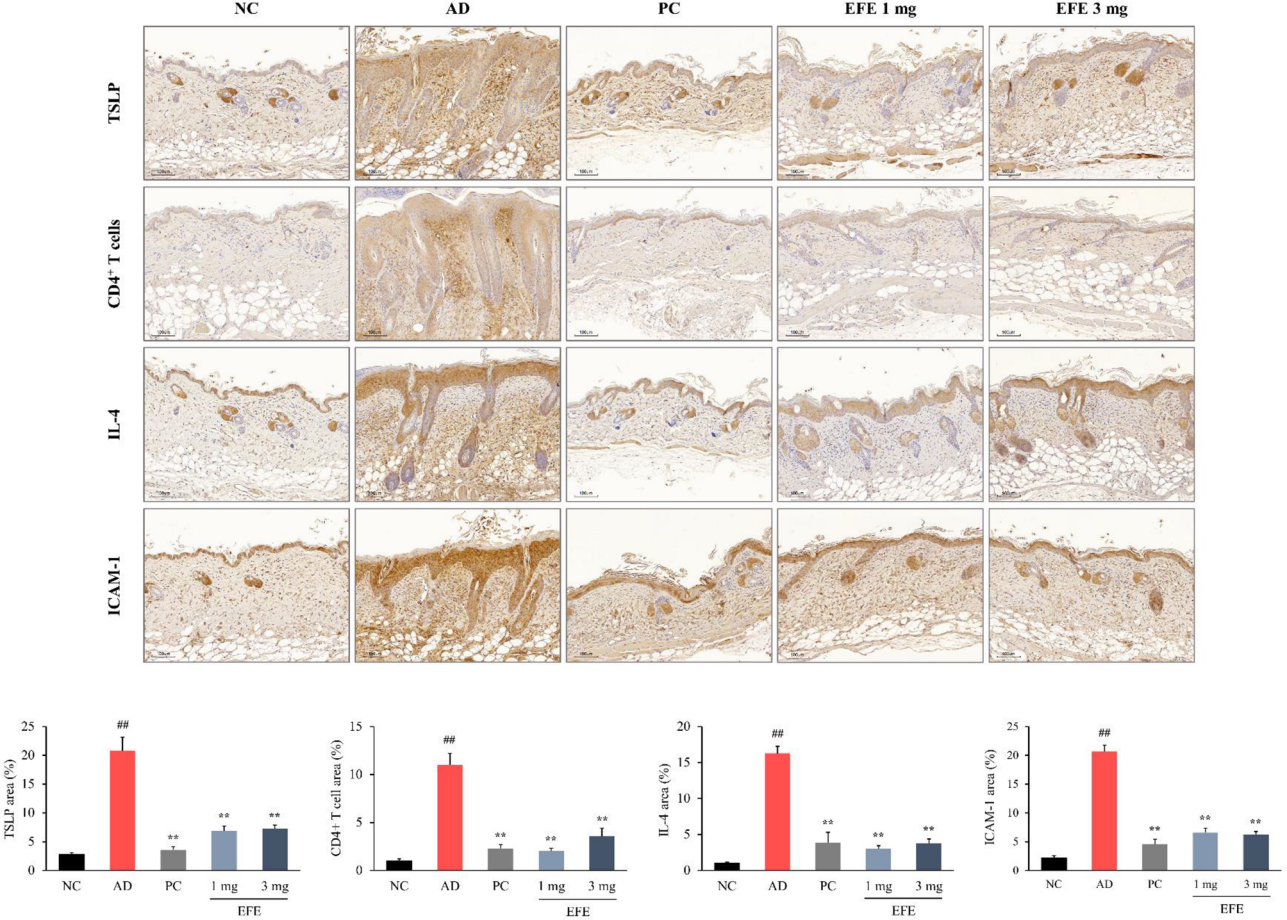
Immunohistochemical analysis of dorsal skin lesions in HDM-induced AD mice. Immunohistochemical staining with antibodies identified TSLP, CD4^+^ T cells, IL-4, and ICAM-1 expression in dorsal skin. Positively stained cells are indicated by dark brown areas. A quantitative morphometric analysis of the immunostained region in relation to the total area was performed. Data are presented as mean ± SEM (n = 7–8). ^##^*p* < 0.01, significantly different from the NC; **p < 0.01, significantly different from the AD. Scale bar = 100 μm. *AD* atopic dermatitis, *EFE* Evodiae Fructus 70% ethanol extract, *HDM* house dust mite, *NC* normal control, *PC* positive control.

IHC staining for TSLP, CD4^+^ T cells, IL-4, and ICAM-1 showed a number of positive reactions in the epidermis and dermis of the AD group compared to the NC group (*p* < 0.01; Fig. 5). In contrast, topical application of prednisolone and EFE (1 and 3 mg) markedly reduced the expression of TSLP, CD4^+^ T cells, IL-4, and ICAM-1 in skin lesions compared to that in the AD group (*p* < 0.01).

### Gene expression profiling of EFE in HaCaT cells

**Figure 6:**
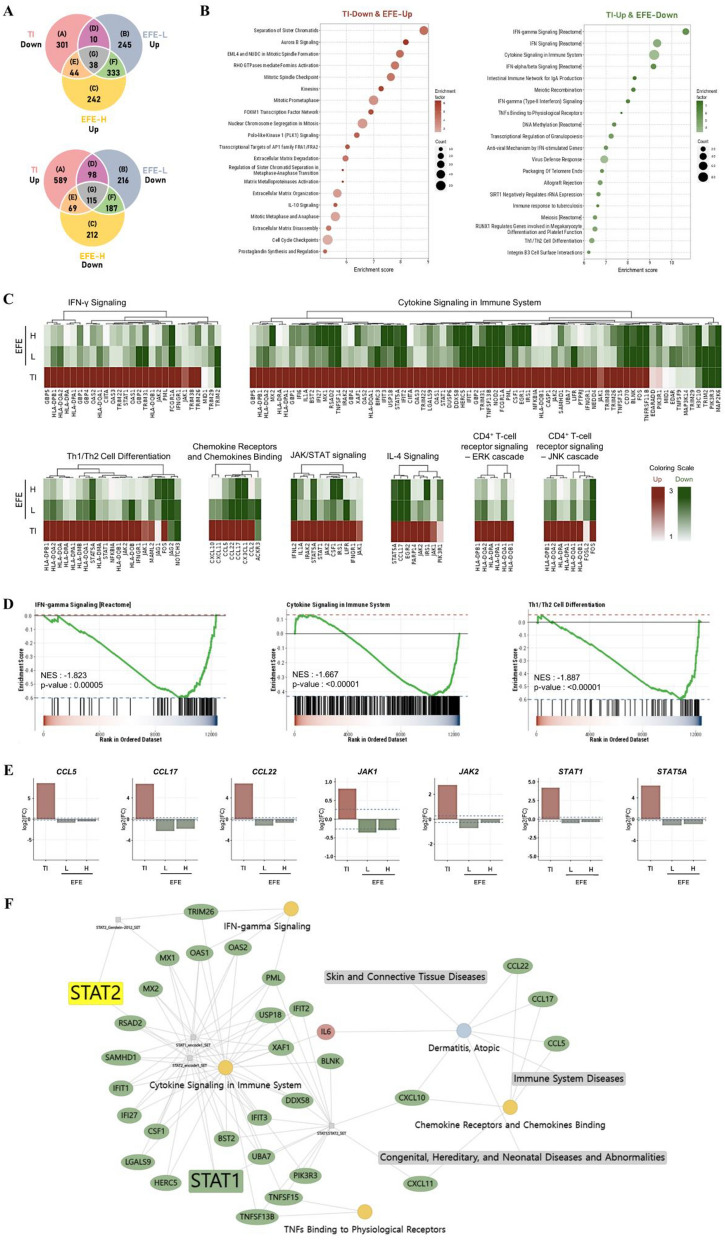
Transcriptomic comparison between TI and EFE-L or H. (**A**) Venn diagram of DEGs for TI and EFE-L or -H. Venn diagrams represent the overlapping and different genes between TI and EFE-L and -H. (**B**) Dotplot of the top 20 enrichment pathways from over-representation analysis. The size of dots represents the count of the gene, and the color of the dots represents its significance. The red and green colors represent up-regulated and down-regulated DEGs compared to TI, respectively. (**C**) Heatmap for DEGs related to significantly enriched pathways between TI and EFE-L or -H. The red and green colors represent up-regulated and down-regulated DEGs, respectively. (**D**) Gene set enrichment analysis of the IFN-γ signaling, cytokine signaling in immune system, and Th1/Th2 cell differentiation-related pathway. (**E**) The gene expression of chemokines (*CCL5*, *CCL17*, and *CCL22*), *JAKs*, and *STATs*. (**F**) Correlation network of the gene-pathway-transcription factor-disease reversed by EFE treatment. Data are expressed as mean (n = 3). *DEGs* differentially expressed genes, *EFE* Evodiae Fructus 70% ethanol extract, *EFE-L* EFE 2.5 μg/mL, *EFE-H* EFE 10 μg/mL, *NC* normal control, *NES* normalized enrichment score, *ORA* over-representation analysis, *RNA-Seq* RNA-sequencing, *TI* TNF-α (10 ng/mL) and IFN-γ (10 ng/mL).

We used RNA-seq to compare the transcriptomes of TI and EFE, which helped identify the MoA of EFE. In TI-treated HaCaT cells, 1264 differentially expressed genes (DEGs) were identified, including 871 up-regulated genes and 393 down-regulated genes compared to NC (Fig. 6A and Supplementary Materials-DEGs list). Venn diagrams in Fig. 6A represent the overlapping as well as different genes between TI and EFE-low (2.5 μg/mL, EFE-L) or high (10 μg/mL, EFE-H) concentrations. The list of genes included in each region is presented in the Supplementary Materials-DEGs list. EFE-L treatment resulted in 48 up-regulated and 213 down-regulated DEGs as compared to TI. The comparison of EFE-H versus TI identified 82 up-regulated and 184 down-regulated DEGs were identified (Fig. 6A). In order to investigate the potential pathways involved in the anti-AD effect of EFE, GSEA was performed using KaiPharm’s database. The top 20 enrichment pathways were presented as dotplots. EFE primarily participated in pathways linked to *IFN-γ signaling*, *cytokine signaling in immune system*, and *Th1/Th2 cell differentiation* (Fig. 6B). Thereafter, the expression patterns of DEGs between TI and EFE were presented as a heatmap of hierarchical clustering analysis (Fig. 6C). The gene sets with *IFN-γ signaling*, *cytokine signaling in immune system*, and *Th1/Th2 cell differentiation* was altered using EFE treatment (Fig. 6D). In particular, AD-associated genes, including *CCL5*, *CCL17*, *CCL22*, *JAK1*, *JAK2*, *STAT1*, and *STAT5A*, were down-regulated by EFE treatment (Fig. 6E). The gene-pathway-transcription factor-disease complex network revealed that EFE regulated *STAT1* and *STAT2* associated with AD (Fig. 6F). Notably, *STAT1* was downregulated by itself as well as by related genes.

### Effect of EFE and its compounds on the JAK-STAT and MAPK signaling pathways in HaCaT cells

**Figure 7:**
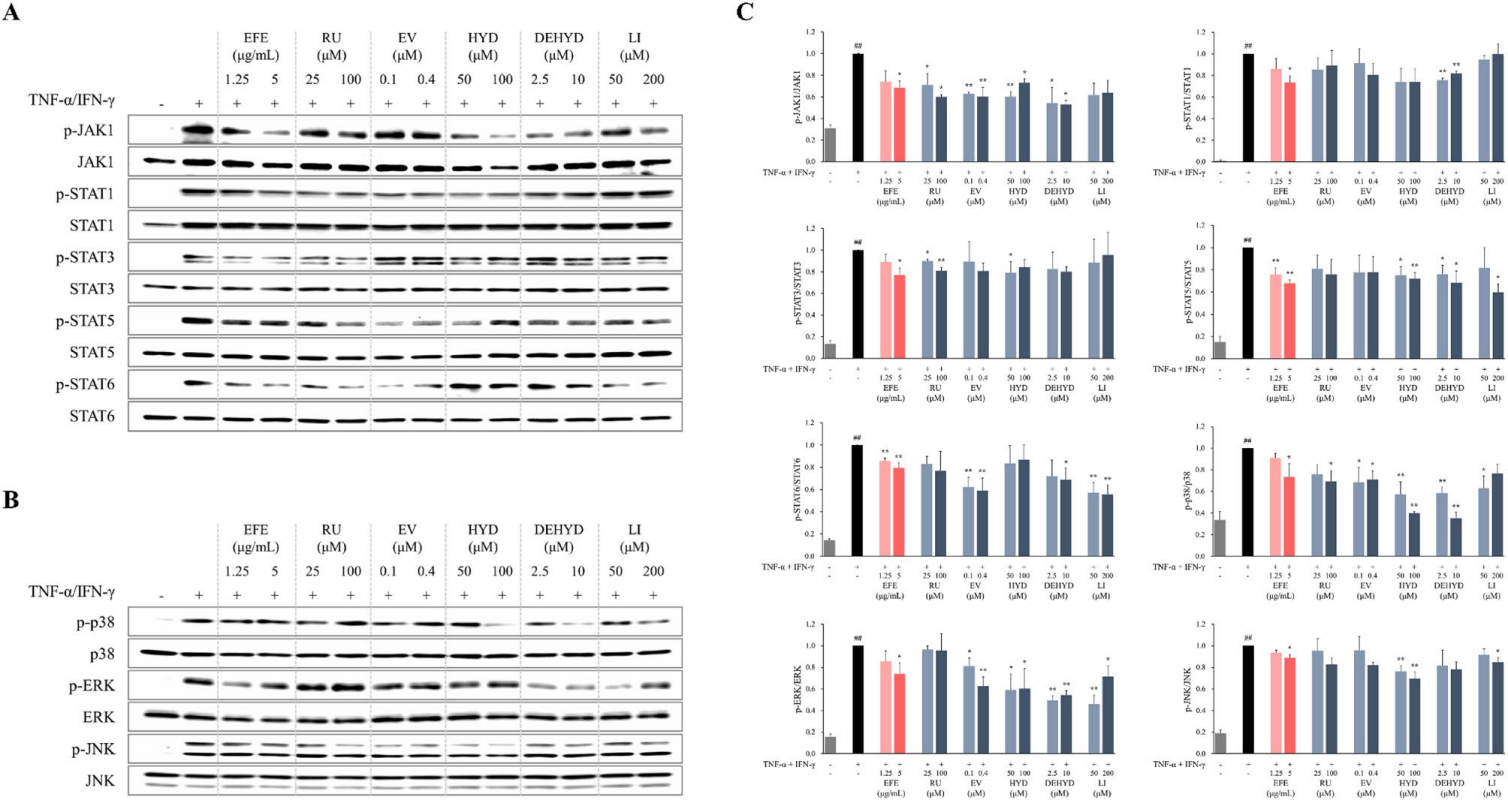
Effect of EFE and its constituents on the activation of JAK-STAT (**A**) and MAPK (**B**) in TI-stimulated HaCaT cells. The cells were treated with EFE and its compounds, and stimulated with TI for 30 min. Expression of total and phosphorylated JAKs and STATs in the cell lysate was determined by Western blotting. (**C**) Data are presented as mean ± SEM as the relative expression ratio of the phosphorylated form to the total form (n = 3). ^#^*p* < 0.05 and ^##^*p* < 0.01 versus NC; *p < 0.05 and **p < 0.01 versus TI-stimulated cells. *DEHYD* dehydroevodiamine, *EFE* Evodiae Fructus 70% ethanol extract, *EV* evodiamine, *HYD* hydroxyevodiamine, *JAK* Janus kinase, *LI* limonin, *NC* normal control, *p-* phosphorylated, *RU* rutaecarpine, *STAT* signal transducers and activators of transcription, *TI* TNF-α (10 ng/mL) and IFN-γ (10 ng/mL).

To explore the potential MoA of EFE in treating AD, the protein expressions in the JAK, STAT, and MAPK signaling pathways were detected using Western blot. The expressions of phosphorylated (p-) JAK1, STATs (STAT1, STAT3, STAT5, and STAT6), and MAPKs (p38, ERK, and JNK) were significantly increased after TI treatment in HaCaT cells (*p* < 0.01; Fig. 7). In contrast, EFE treatment greatly decreased the phosphorylation of JAK1, STATs including STAT1, STAT3, STAT5, and STAT6, and MAPKs including p38, ERK, and JNK (*p* < 0.05 or *p* < 0.01). We investigated whether the compounds contained in EFE affect the JAK-STAT and MAPK signaling pathways and found that rutaecarpine, evodiamine, hydroxyevodiamine, and dehydroevodiamine significantly inhibited the JAK1 activation (*p* < 0.05 or *p* < 0.01). Though limonin reduced JAK1 phosphorylation, the difference was not statistically significant. Dehydroevodiamine significantly inhibited STAT1 phosphorylation, whereas rutaecarpine and hydroxyevodiamine markedly reduced STAT3 phosphorylation (*p* < 0.05 or *p* < 0.01). Hydroxyevodiamine, dehydroevodiamine, and limonin downregulated the expression of p-STAT5 (*p* < 0.05 or *p* < 0.01), and evodiamine, dehydroevodiamine, and limonin exerted marked inhibition on STAT6 activation (*p* < 0.05 or *p* < 0.01). All five compounds significantly reduced the expression of p-p38 (*p* < 0.05 or *p* < 0.01). Except for rutaecarpine, the other four compounds suppressed ERK activation (*p* < 0.05 or *p* < 0.01). The expression of p-JNK was greatly reduced by hydroxyevodiamine and limonin (*p* < 0.05 or *p* < 0.01).

## Discussion

In this study, EFE significantly inhibited inflammatory responses in the in vitro model using TI-stimulated HaCaT cells and in the in vivo AD model using HDM-treated NC/Nga mice.

The effects of natural products on disease management may result from the combined action of various compounds^24^. First, we identified nine constituents of EFE using LC–MS/MS; rutaecarpine (Comp.1), evodiamine (Comp.2), evodol (Comp.3), dehydroevodiamine (Comp.4), limonin (Comp.5), synephrine (Comp.6), evocarpine (Comp.7), dihydroevocarpine (Comp.8), and hydroxyevodiamine (Comp.9). In a previous study, Evodiae Fructus and its main constituents, rutaecarpine and evodiamine, were reported to suppress the scratching behaviors in compound 48/80-induced passive cutaneous anaphylaxis reaction^13^. Furthermore, rutaecarpine improves imiquimod-induced psoriasis-like dermatitis by modulating the nuclear factor kappa-light-chain-enhancer of activated B cells (NF-κB) and toll-like receptor 7 signaling^25^. Although there is an article in Chinese stating that rutaecarpine reduces plasma IL-4, IgE, and IFN-γ levels in the 2,4-dinitrochlorobenzene-induced eczematous dermatitis model, the dermatitis alleviating effect and detailed mechanism have not been confirmed^26^. Additionally, some studies have shown that limonin improves picryl chloride-induced contact hypersensitivity^27^. Considering the biological functions of these components, it is likely that EFE could act on multiple AD-related therapeutic targets.

The epidermis, the skin’s outer layer, comprises keratinocytes at various phases of differentiation and functions as a barrier against external agents such as antigens and pathogens. TSLP secreted by keratinocytes stimulates the production of Th2-attracting chemokines^11,28^, which play important roles in AD pathogenesis by attracting immune cells to the skin^29^. Epidermis-derived RANTES is a key mediator in the recruitment of Langerhans cells which play a crucial role in the initiation and regulation of immune responses to the epidermis^30^. RANTES has been reported to be elevated in the skin lesions of AD patients, implying that it is involved in the pathogenesis of AD^31^. TARC and MDC also have been reported to be significantly associated with AD^32^. In this study, HaCaT cells were stimulated with TI using an in vitro model based on the molecular level of AD, as described in a previous study^19^. Our results showed that EFE reduced RANTES, TARC, and MDC in TI-stimulated HaCaT cells, which could be a result of the multiple effects of compounds, such as rutaecarpine, evodiamine, hydroxyevodiamine, dehydroevodiamine, and limonin.

Herbal medicines exert their holistic effects through the synergistic or additive action of the multi-compounds^33^. Rutaecarpine, evodiamine, and dehydroevodiamine, the major components abundantly present in EFE, belong to alkaloids known to inhibit the JAK-STAT pathway^34^. Although the amount of limonin in EFE is relatively less than those of the other three major alkaloids in EFE, it is one of the terpenoids that suppresses the phosphorylation of JAK-STAT^34^. At EFE 10 μg/mL, which inhibits the release of chemokines in TI-stimulated HaCaT cells, the content of evodiamine can be quantified at 0.09 μg/mL. It corresponds to approximately 73% of the maximum treatment concentration of evodiamine (0.4 μM = 0.12 μg/mL), suggesting that evodiamine may contribute most to the anti-atopic dermatitis effects of EFE. Similarly, the concentrations of rutaecarpine (0.07 μg/mL), hydroxyevodiamine (0.0001 μg/mL), dehydroevodiamine (0.08 μg/mL), and limonin (0.03 μg/mL) contained in EFE at 10 μg/mL were lower than the effective concentrations of each component (rutaecarpine 100 μM = 28.73 μg/mL; hydroxyevodiamine 100 μM = 31.94 μg/mL; dehydroevodiamine 10 μM = 3.01 μg/mL; limonin 200 μM = 94.10 μg/mL). Therefore, the combination of other multi-compounds, with evodiamine as the main active ingredient, can be considered to synergistically contribute to efficacy of EFE.

Based on the in vitro findings, the effect of EFE on HDM-treated NC/Nga mice was studied to evaluate whether EFE exhibited an AD therapeutic effect in vivo. The EFE dose was established at 3 mg/mouse, which is comparable to 2%, and 1 mg/mouse, which is 1/3, based on the content of topical ointments used clinically^35^. In HDM-treated mice, various clinical symptoms similar to those of patients with AD, such as erythema/hemorrhage, edema, excoriation/erosion, and scaling/dryness, were clearly observed in the dorsal skin and ear but were alleviated by treatment with EFE. Additionally, the topical application of EFE improved inflammatory cell infiltration and epidermal thickening, including hyperkeratosis and hyperplasia of the epithelium, as shown by H&E and TB staining. The release of plasma histamine but not total IgE was significantly reduced by EFE treatment. These results suggest that EFE reduces histamine levels by inhibiting mast cell activation in association with reduced mast cell infiltration in skin lesions, but does not affect systemic total IgE levels. Prednisolone, used as a positive control, had the effect of reducing plasma histamine and IgE levels but had the side effect of decreasing body weight and skin atrophy compared to normal controls. This is in contrast to EFE and suggests the possibility that EFE can be applied more safely than prednisolone.

The spleen contains a variety of immune cells and plays a key role in regulating the immune system. Therefore, an increase in immune function may cause splenomegaly^36^. As a result of observing the characteristics of the spleen in the AD model, the spleen index was significantly increased in the AD group compared to that in the normal control group, and decreased to a level similar to that of the normal control with EFE treatment. Our results suggest that EFE reduces splenic enlargement by restoring aberrant immune system functions. However, the prednisolone group had smaller spleens than the normal control group, suggesting that there may be severe immunosuppressive side effects due to a remarkable reduction in the spleen index.

TSLP-stimulated dendritic cells prime naïve CD4^+^ T cells to generate cytokines such as IL-4, -5, -13, and TNF-α. To verify the relationship between these mediators, we evaluated TSLP, CD4^+^ T cells, and IL-4 expression using IHC. According to our findings, EFE treatment significantly reduced HDM-induced elevation of TSLP, CD4^+^ T cells, and IL-4. Adhesion molecules are critical for the homing of T cell subsets to skin lesions in patients with AD. Among them, intercellular adhesion molecule-1 (ICAM-1) has been reported to be highly expressed in skin lesions of AD patients and to play an important role in the pathogenesis of AD^37^. In our AD mice, EFE treatment markedly decreased the expression of ICAM-1, which was increased in lesional skin by HDM. Our finding that EFE, herbal medicine, is beneficial in improving atopic dermatitis, even at a content of less than 2%, is regarded as a meaningful outcome compared to the content of ointments used clinically.

Stress causes the release of several hormones, including corticosterone^38^. In this study, plasma corticosterone levels were measured to determine whether AD alleviation by EFE treatment resulted in changes in psychological stress. As a result, plasma corticosterone levels elevated by stress in HDM-induced AD mice were significantly reduced by EFE treatment. Evodiamine contained in EFE has been reported to have an antidepressant-like effect by modulating central monoamine neurotransmitters and corticosterone^39^, suggesting that this may contribute to the stress hormone-restoring effect of EFE.

Transcriptomic analysis in HaCaT cells revealed that the major pathway by which EFE targeted AD treatment involved the JAK-STAT signaling pathway. The activated JAKs phosphorylate the residues on cytokine receptors to recruit STAT proteins^40^. STAT is phosphorylated by JAK, following which it is dimerized and translocated to the nucleus to induce the expression of cytokine-related genes^40^. Alterations of the JAK-STAT signaling pathway are implicated in the pathogenesis of AD and are involved in Th2 immunological polarization, eosinophil activation, and skin barrier dysfunction during AD progression^41^. Therefore, we verified whether EFE in HaCaT cells can reduce AD-like skin inflammation by down-regulating the JAK-STAT signaling pathway. We found that EFE down-regulated the expression of p-JAK1 and p-STATs (p-STAT1, STAT3, STAT5, and STAT6) in TI-stimulated HaCaT cells, which was consistent with the findings of transcriptome analysis. Rutaecarpine, evodiamine, hydroxyevodiamine, dehydroevodiamine, and limonin contained in EFE selectively suppressed phosphorylation of JAK1 and STAT1, STAT3, STAT5, and STAT6. Our findings suggest that the anti-AD action of EFE is related to the JAK-STAT pathway, which can be attributed to the active ingredients contained in EFE.

The JAK-STAT pathways also activate the MAPKs, whose activation produces inflammatory cytokines and plays a central role in the progression of AD^42,43^. In the present study, we found that EFE and its compounds suppressed MAPK phosphorylation in TI-stimulated HaCaT cells. Hence, EFE likely down-regulates the activation of the MAPK signaling pathway via selective inhibition of p38, ERK, and JNK by multiple compounds, which contributes to the anti-AD action of EFE. In the future, the findings of the present study can aid in identifying the correlation between the structure and MoA of components contained in EFE.

## Conclusions

In summary, our findings suggest that EFE inhibited the production of inflammatory mediators by down-regulating the activation of the JAK-STAT and MAPK signaling pathways in keratinocytes and alleviating AD symptoms in vivo*.* It was also revealed that rutaecarpine, evodiamine, hydroxyevodiamine, dehydroevodiamine, and limonin contained in EFE acted as major active compounds for anti-AD. Our results indicate that EFE can alleviate the complex pathophysiology of AD through various active compounds that affect multiple inflammatory mediators.

### Supplementary Information

Supplementary Figures.

Supplementary Information 1.

Supplementary Information 2.

## Data Availability

All data generated or analyzed during this study are included in this published article (and its Supplementary Information files).

## References

[1] Kabashima K (2013). New concept of the pathogenesis of atopic dermatitis: Interplay among the barrier, allergy, and pruritus as a trinity. J. Dermatol. Sci..

[2] Ohmori K (2014). Time-of-day-dependent variations of scratching behavior and transepidermal water loss in mice that developed atopic dermatitis. J. Vet. Med. Sci..

[3] Boguniewicz M, Leung DY (2011). Atopic dermatitis: A disease of altered skin barrier and immune dysregulation. Immunol. Rev..

[4] Torres T (2019). Update on atopic dermatitis. Acta Med. Port..

[5] Cho K (2018). *Pyrus*
*ussuriensis* Maxim. leaves extract ameliorates DNCB-induced atopic dermatitis-like symptoms in NC/Nga mice. Phytomedicine.

[6] Lee JH, Jeon YD, Lee YM, Kim DK (2018). The suppressive effect of puerarin on atopic dermatitis-like skin lesions through regulation of inflammatory mediators in vitro and in vivo. Biochem. Biophys. Res. Commun..

[7] Han SC (2012). Fermented fish oil suppresses T helper 1/2 cell response in a mouse model of atopic dermatitis via generation of CD4+CD25+Foxp3+ T cells. BMC Immunol..

[8] Choi D, Kang W, Park T (2020). Anti-allergic and anti-inflammatory effects of undecane on mast cells and keratinocytes. Molecules.

[9] Galli SJ, Tsai M (2012). IgE and mast cells in allergic disease. Nat. Med..

[10] Wang L (2021). Efficacy and action mechanisms of a Chinese herbal formula on experimental models of atopic dermatitis. J. Ethnopharmacol..

[11] Jia Y (2023). Hydrogel dressing integrating FAK inhibition and ROS scavenging for mechano-chemical treatment of atopic dermatitis. Nat. Commun..

[12] Xia H (2023). Chromatographic and mass spectrometric technologies for chemical analysis of Euodiae fructus: A review. Phytochem. Anal..

[13] Shin YW, Bae EA, Cai XF, Lee JJ, Kim DH (2007). In vitro and in vivo antiallergic effect of the fructus of *Evodia*
*rutaecarpa* and its constituents. Biol. Pharm. Bull..

[14] Yarosh DB (2006). Anti-inflammatory activity in skin by biomimetic of *Evodia*
*rutaecarpa* extract from traditional Chinese medicine. J. Dermatol. Sci..

[15] Matsuda H, Wu JX, Tanaka T, Iinuma M, Kubo M (1997). Antinociceptive activities of 70% methanol extract of evodiae fructus (fruit of *Evodia*
*rutaecarpa* var. *bodinieri*) and its alkaloidal components. Biol. Pharm. Bull..

[16] Zhao Y, Zhao Y, Zhou X, Gong X (2014). Development and validation of an UPLC-ESI-MS/MS method for determination of dehydroevodiamine, limonin, evodiamine, and rutaecarpine in Evodiae Fructus. Pharmacogn. Mag..

[17] Li M, Wang C (2020). Traditional uses, phytochemistry, pharmacology, pharmacokinetics and toxicology of the fruit of *Tetradium*
*ruticarpum*: A review. J. Ethnopharmacol..

[18] Jin SE (2015). Traditional herbal formula Banhasasim-tang exerts anti-inflammatory effects in RAW 264.7 macrophages and HaCaT keratinocytes. Evid. Based Complement Altern. Med..

[19] Jin SE, Ha H, Shin HK, Seo CS (2019). Anti-allergic and anti-inflammatory effects of Kuwanon G and Morusin on MC/9 mast cells and HaCaT keratinocytes. Molecules.

[20] Yamamoto M (2007). A novel atopic dermatitis model induced by topical application with *Dermatophagoides*
*farinae* extract in NC/Nga mice. Allergol. Int..

[21] Jin SE (2020). Topical application of a new herbal complex, NI-01, ameliorates house dust mite-induced atopic dermatitis in NC/Nga mice. Nutrients.

[22] Hanifin JM (2001). The eczema area and severity index (EASI): Assessment of reliability in atopic dermatitis. EASI Evaluator Group. Exp. Dermatol..

[23] *Guide for the Care and Use of Laboratory Animals, 8th edn*. (National Research Council (US) Committee, 2011).

[24] Xu R (2022). Analysis of the molecular mechanism of *Evodia*
*rutaecarpa* fruit in the treatment of nasopharyngeal carcinoma using network pharmacology and molecular docking. J. Healthc. Eng..

[25] Li Y (2019). Rutaecarpine inhibited imiquimod-induced psoriasis-like dermatitis via inhibiting the NF-kappaB and TLR7 pathways in mice. Biomed. Pharmacother..

[26] Tong M, Guo YN, Zhang GY (2011). Effect and mechanisms of rutaecarpine on treating atopic dermatitis in mice. Sichuan Da Xue Xue Bao Yi Xue Ban.

[27] Wang X (2015). Obaculactone exerts a novel ameliorating effect on contact dermatitis through regulating T lymphocyte. Int. Immunopharmacol..

[28] Soumelis V (2002). Human epithelial cells trigger dendritic cell mediated allergic inflammation by producing TSLP. Nat. Immunol..

[29] Castan L, Magnan A, Bouchaud G (2017). Chemokine receptors in allergic diseases. Allergy.

[30] Ouwehand K (2012). CCL5 and CCL20 mediate immigration of Langerhans cells into the epidermis of full thickness human skin equivalents. Eur. J. Cell Biol..

[31] Morita E, Kameyoshi Y, Hiragun T, Mihara S, Yamamoto S (2001). The C-C chemokines, RANTES and eotaxin, in atopic dermatitis. Allergy.

[32] Jahnz-Rozyk K, Targowski T, Paluchowska E, Owczarek W, Kucharczyk A (2005). Serum thymus and activation-regulated chemokine, macrophage-derived chemokine and eotaxin as markers of severity of atopic dermatitis. Allergy.

[33] Liu LL, Liu Q, Li P, Liu EH (2018). Discovery of synergistic anti-inflammatory compound combination from herbal formula GuGe FengTong Tablet. Chin. J. Nat. Med..

[34] Bose S (2020). Targeting the JAK/STAT signaling pathway using phytocompounds for cancer prevention and therapy. Cells.

[35] Kleinman E, Laborada J, Metterle L, Eichenfield LF (2022). What's new in topicals for atopic dermatitis?. Am. J. Clin. Dermatol..

[36] Chapman, J., Goyal, A. & Azevedo, A. M. in *StatPearls* (2023).

[37] Lugovic L, Cupic H, Lipozencic J, Jakic-Razumovic J (2006). The role of adhesion molecules in atopic dermatitis. Acta Dermatovenerol. Croat..

[38] Amano H, Negishi I, Akiyama H, Ishikawa O (2008). Psychological stress can trigger atopic dermatitis in NC/Nga mice: An inhibitory effect of corticotropin-releasing factor. Neuropsychopharmacology.

[39] Jiang ML (2015). Antidepressant-like effect of evodiamine on chronic unpredictable mild stress rats. Neurosci. Lett..

[40] Nakashima C, Yanagihara S, Otsuka A (2022). Innovation in the treatment of atopic dermatitis: Emerging topical and oral Janus kinase inhibitors. Allergol. Int..

[41] Xue C (2023). Evolving cognition of the JAK-STAT signaling pathway: Autoimmune disorders and cancer. Signal Transduct. Target Ther..

[42] Qinwufeng G (2022). Jiu-Wei-Yong-An Formula suppresses JAK1/STAT3 and MAPK signaling alleviates atopic dermatitis-like skin lesions. J. Ethnopharmacol..

[43] Winston LA, Hunter T (1996). Intracellular signalling: Putting JAKs on the kinase MAP. Curr. Biol..

