# Supplementary material for: Evodiae Fructus extract suppresses inflammatory response in HaCaT cells and improves house dust mite-induced atopic dermatitis in NC/Nga mice
Source: Sci Rep. 2024 Jan 4;14:472. doi: 10.1038/s41598-023-50257-3 (PMC10764943; doi:10.1038/s41598-023-50257-3)

Original Western blot images in triplicate

1st Membrane #1

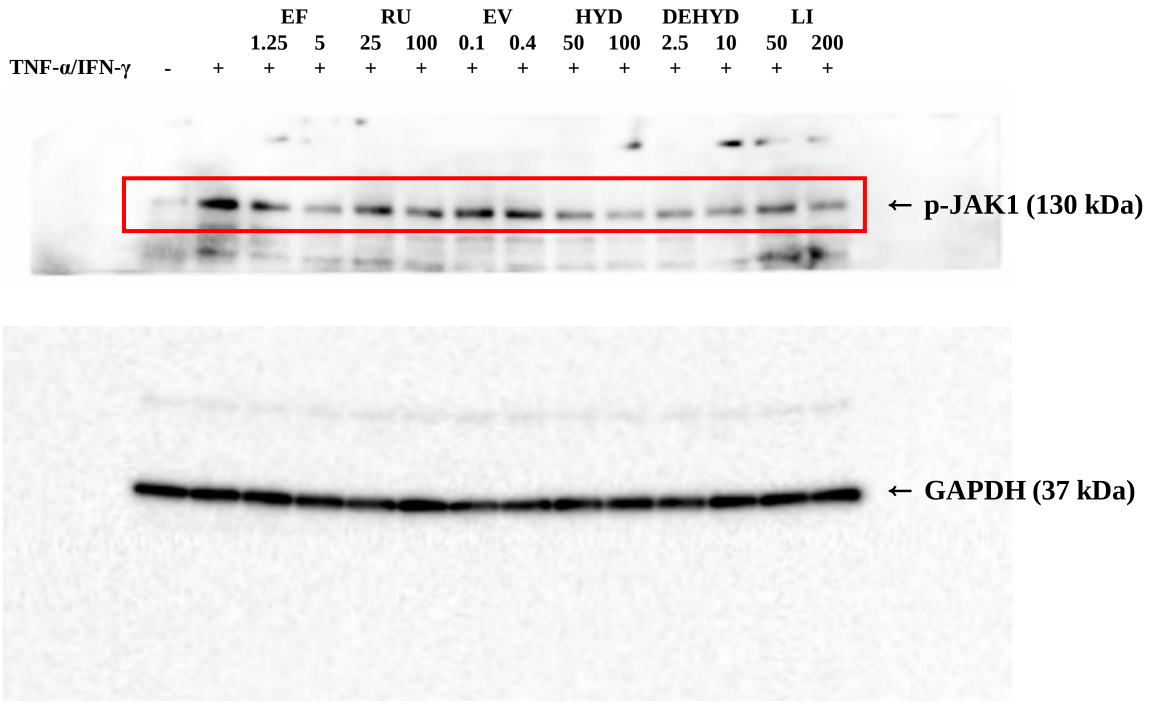

strip the membrane

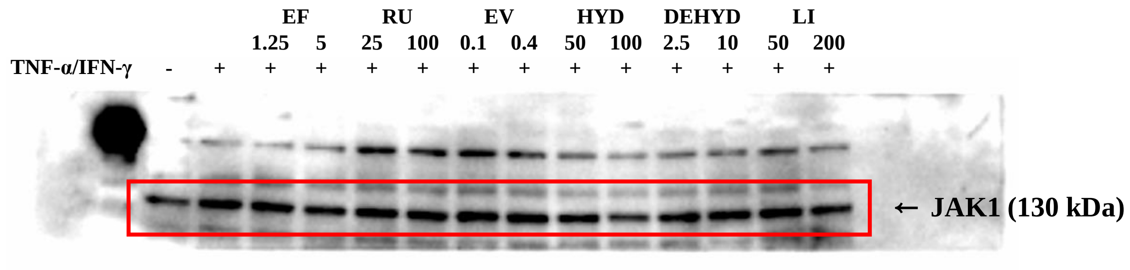

1st Membrane #2

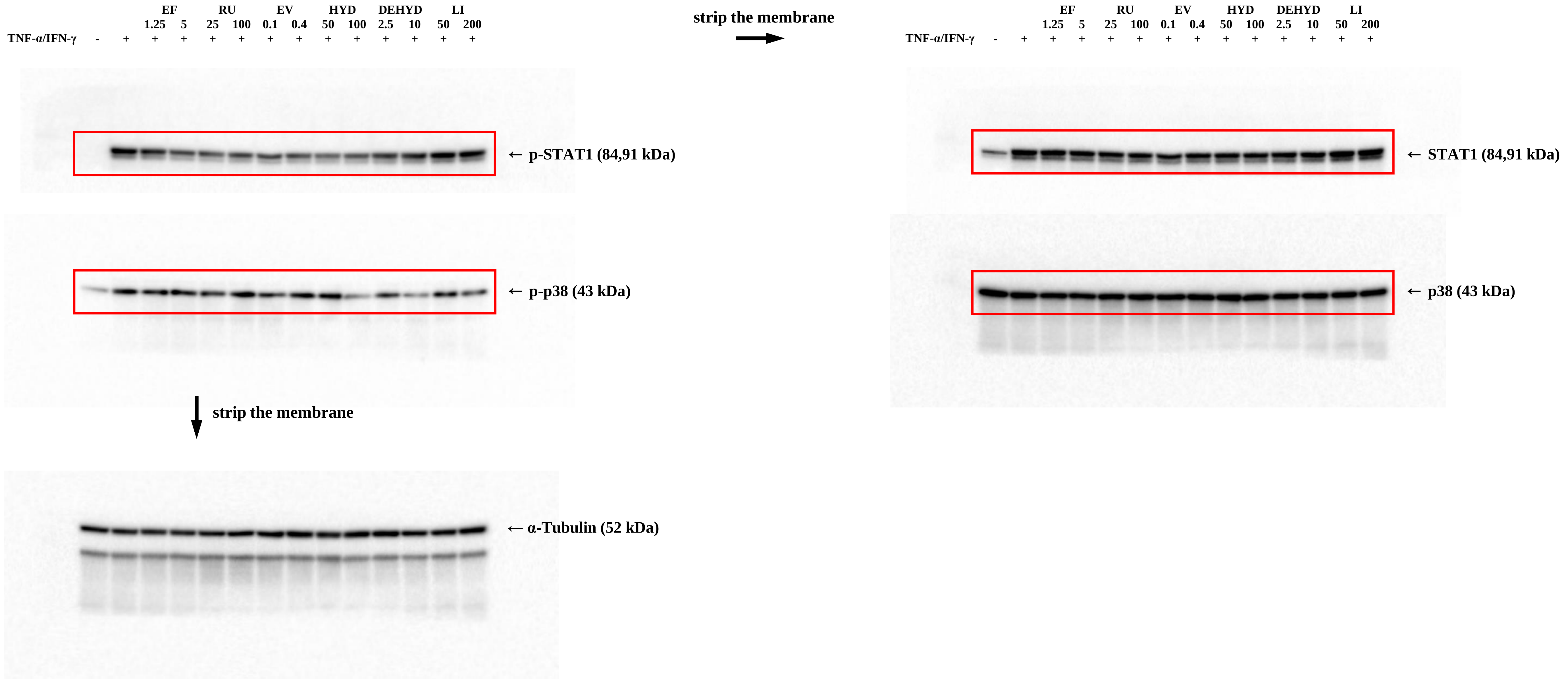

1st Membrane #3

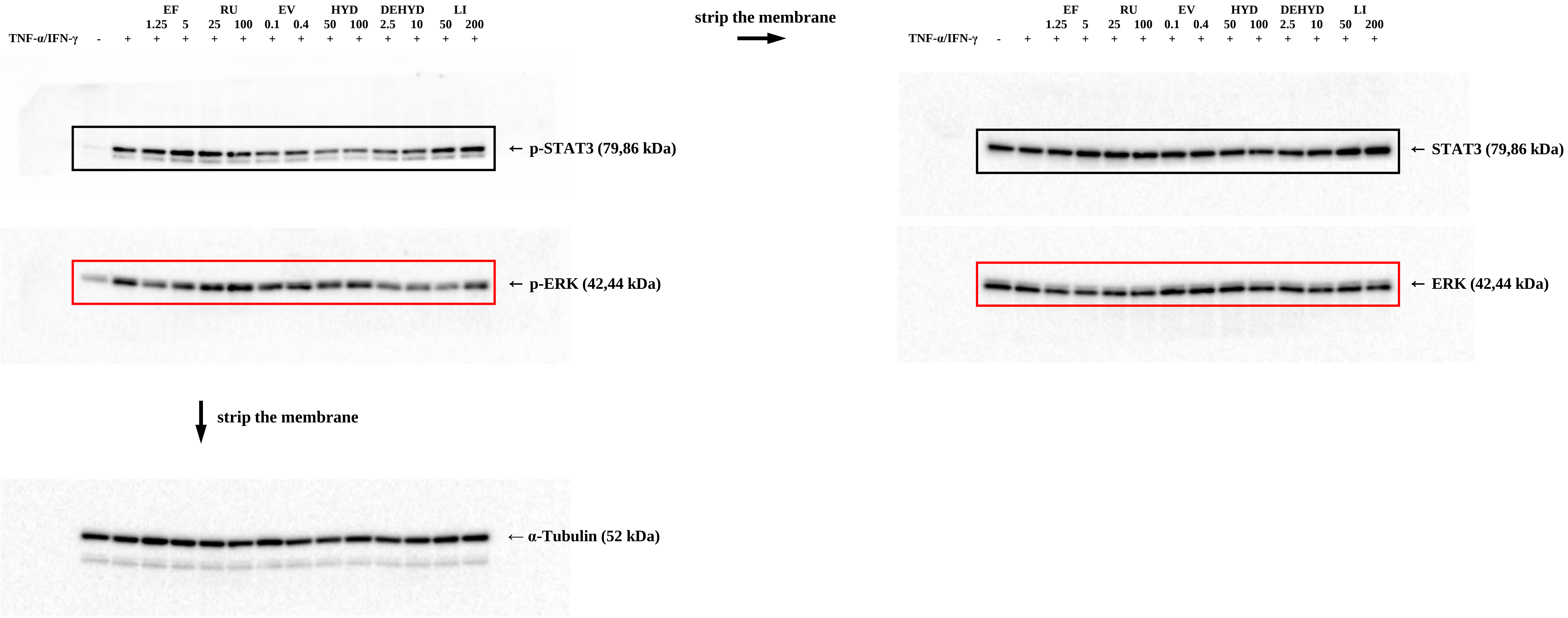

1st Membrane #4

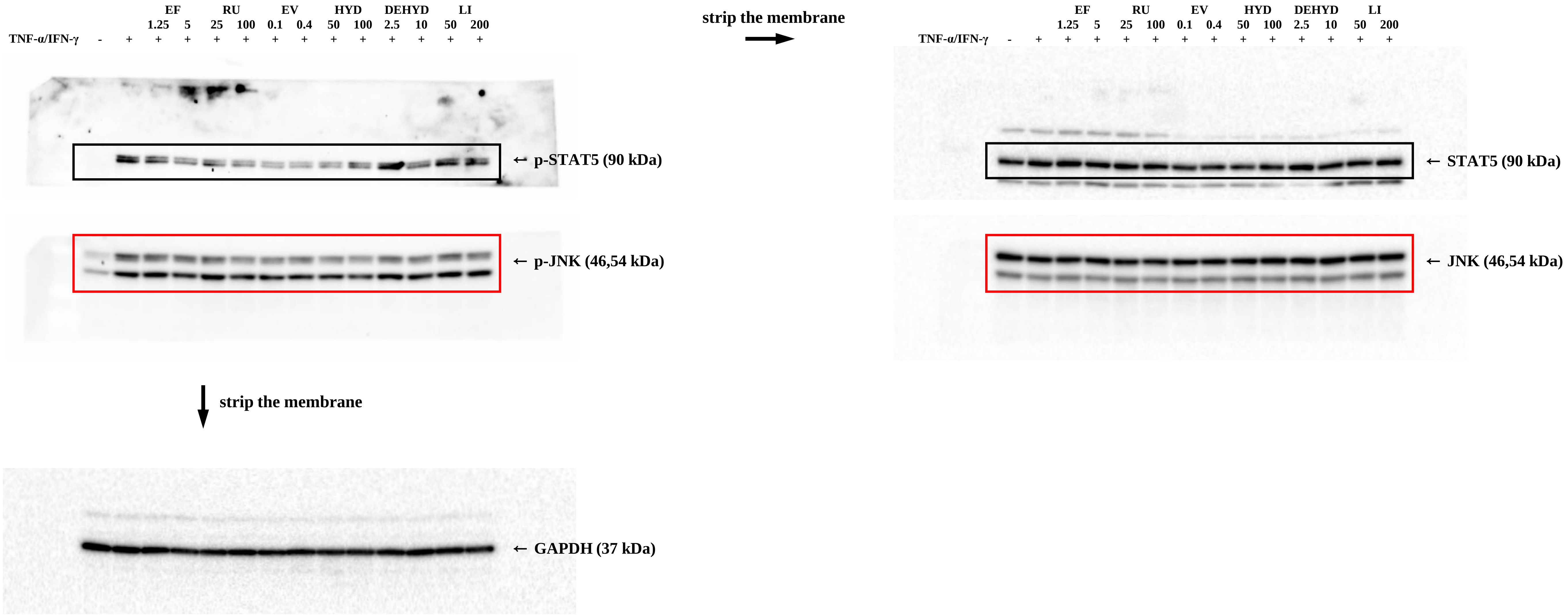

1st Membrane #5

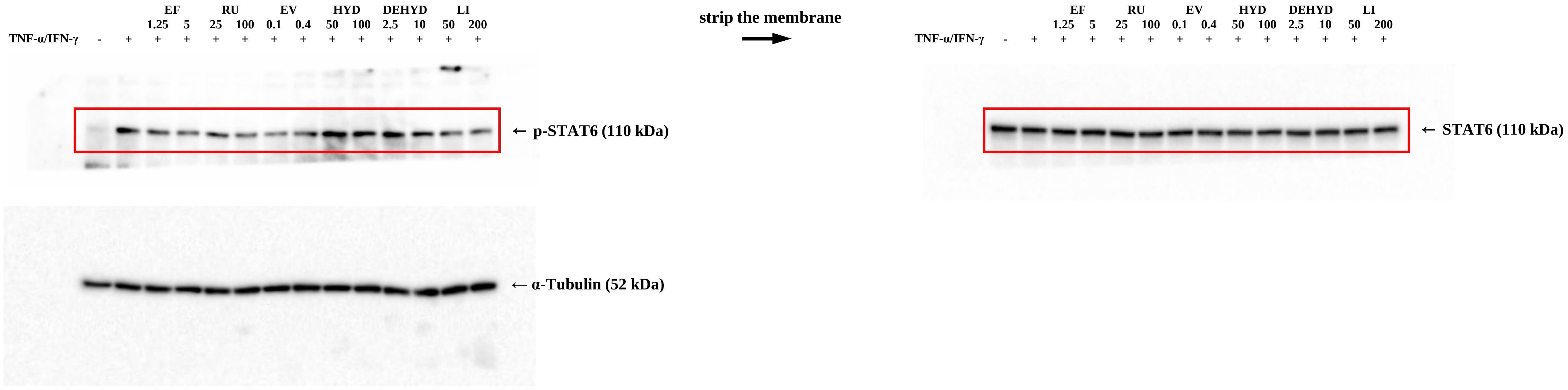

2nd Membrane #1

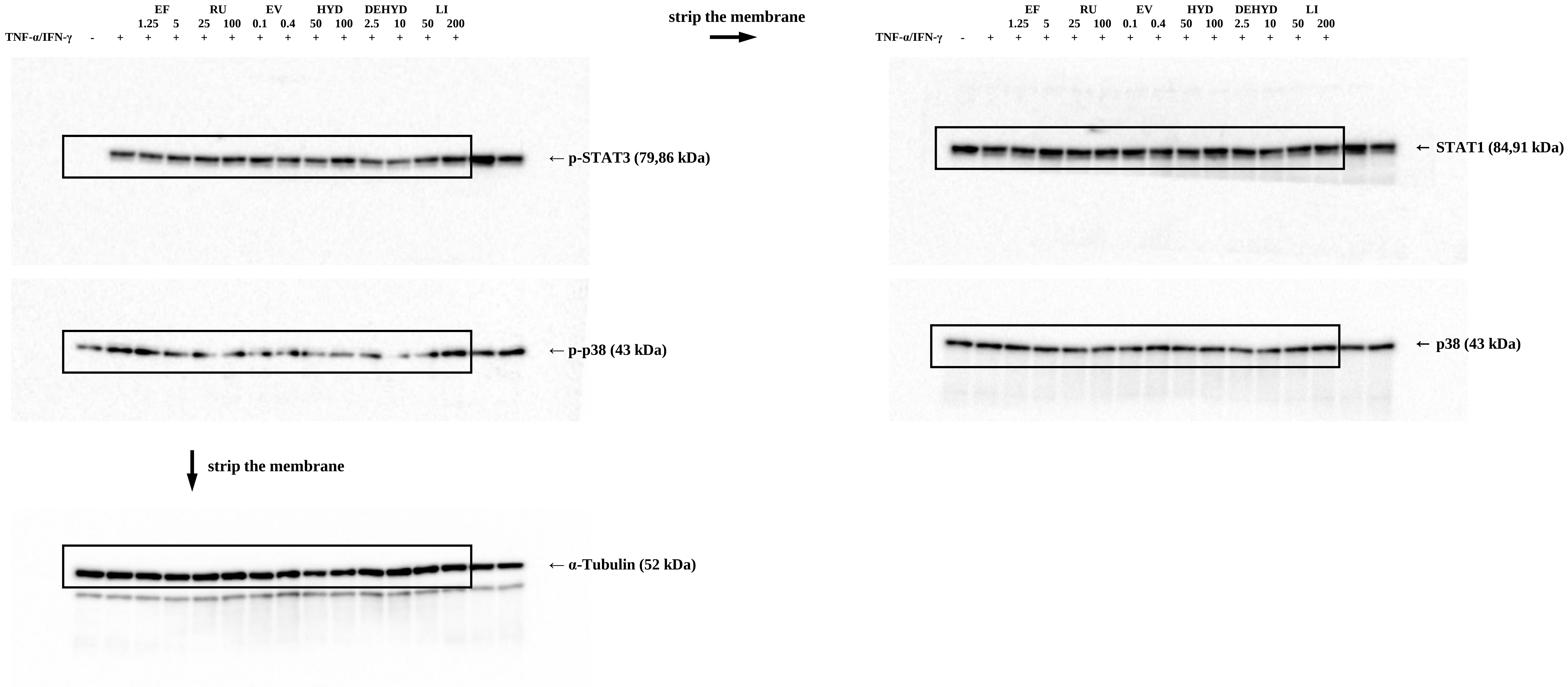

2nd Membrane #2

			EF		RU		EV		HYD		DEHYD		LI	
			1.25	5	25	100	0.1	0.4	50	100	2.5	10	50	200
TNF-α/IFN-γ	-	+	+	+	+	+	+	+	+	+	+	+	+	+

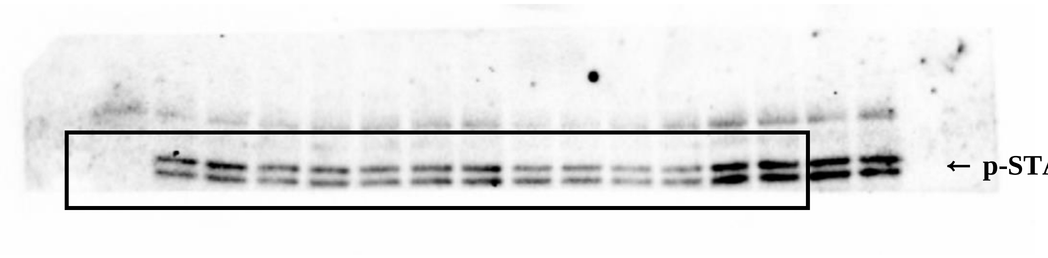

← p-STAT3 (79,86 kDa)

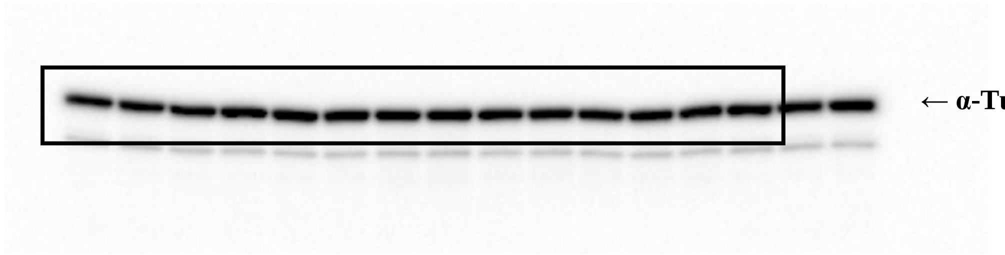

← α-Tubulin (52 kDa)

strip the membrane →

			EF		RU		EV		HYD		DEHYD		LI	
			1.25	5	25	100	0.1	0.4	50	100	2.5	10	50	200
TNF-α/IFN-γ	-	+	+	+	+	+	+	+	+	+	+	+	+	+

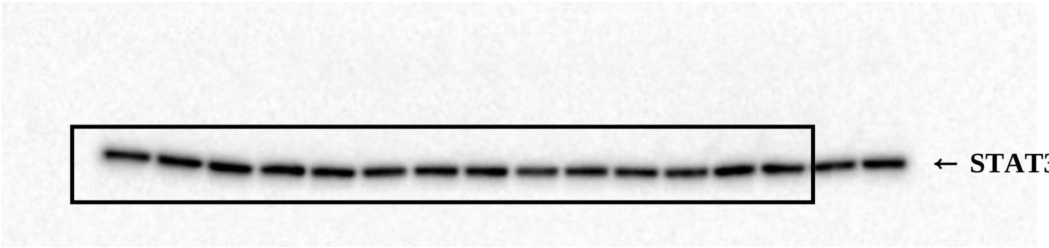

← STAT3 (79,86 kDa)

2nd Membrane #3

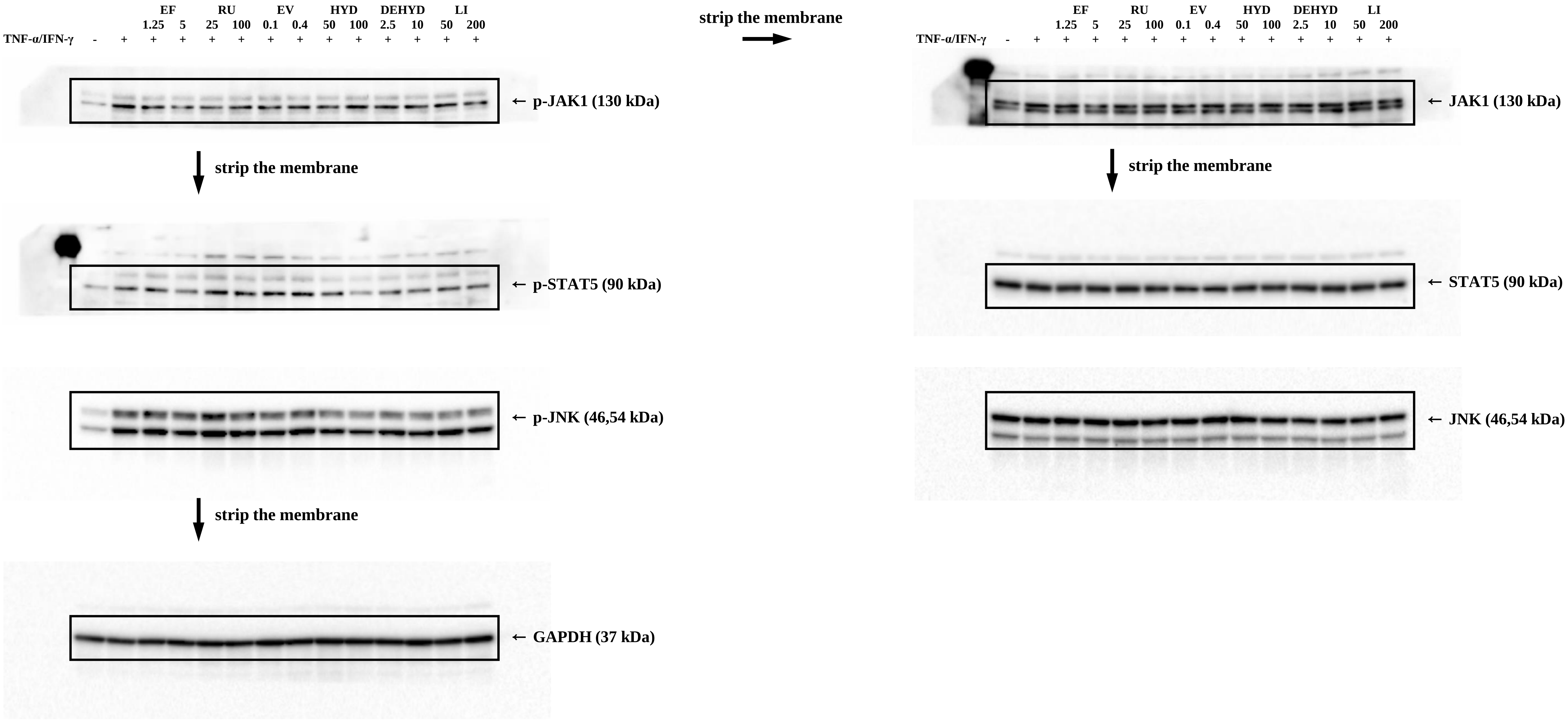

2nd Membrane #4

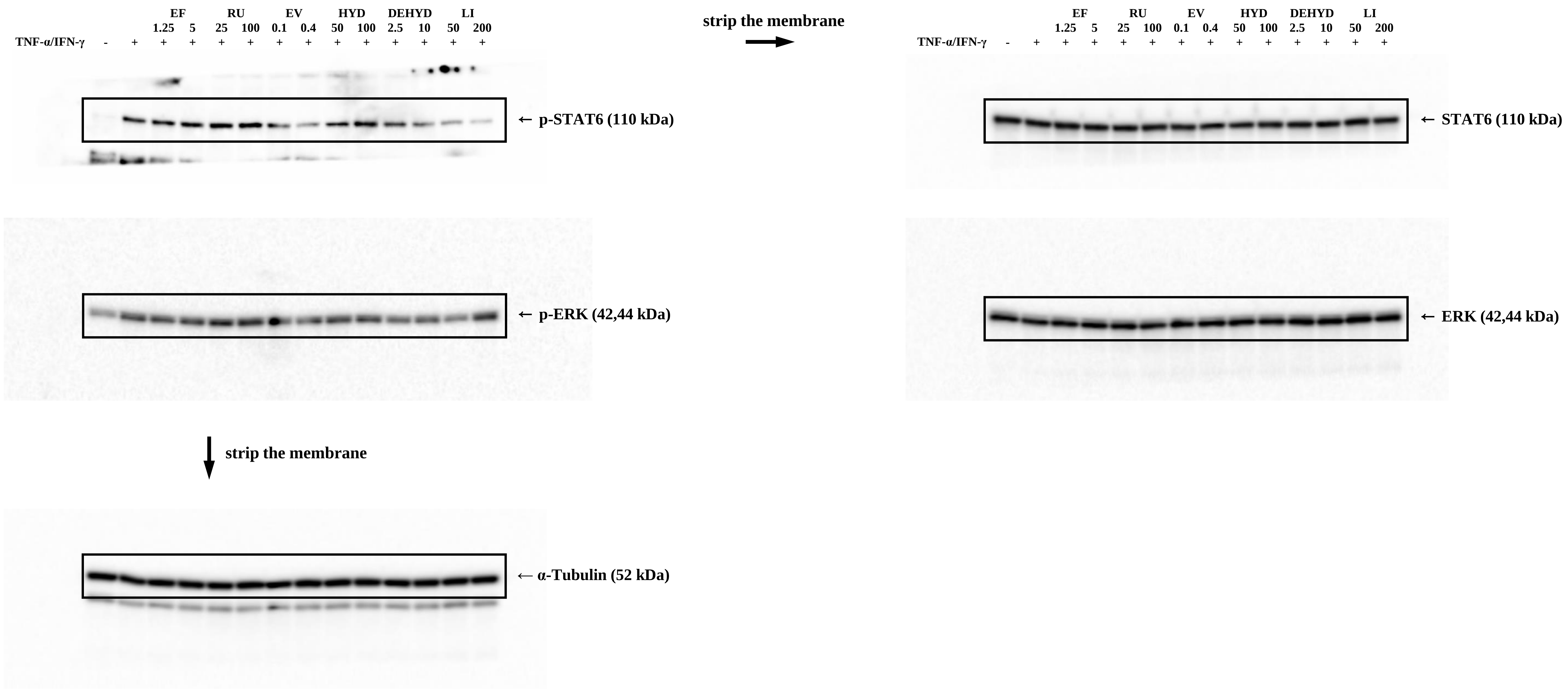

3rd Membrane #1

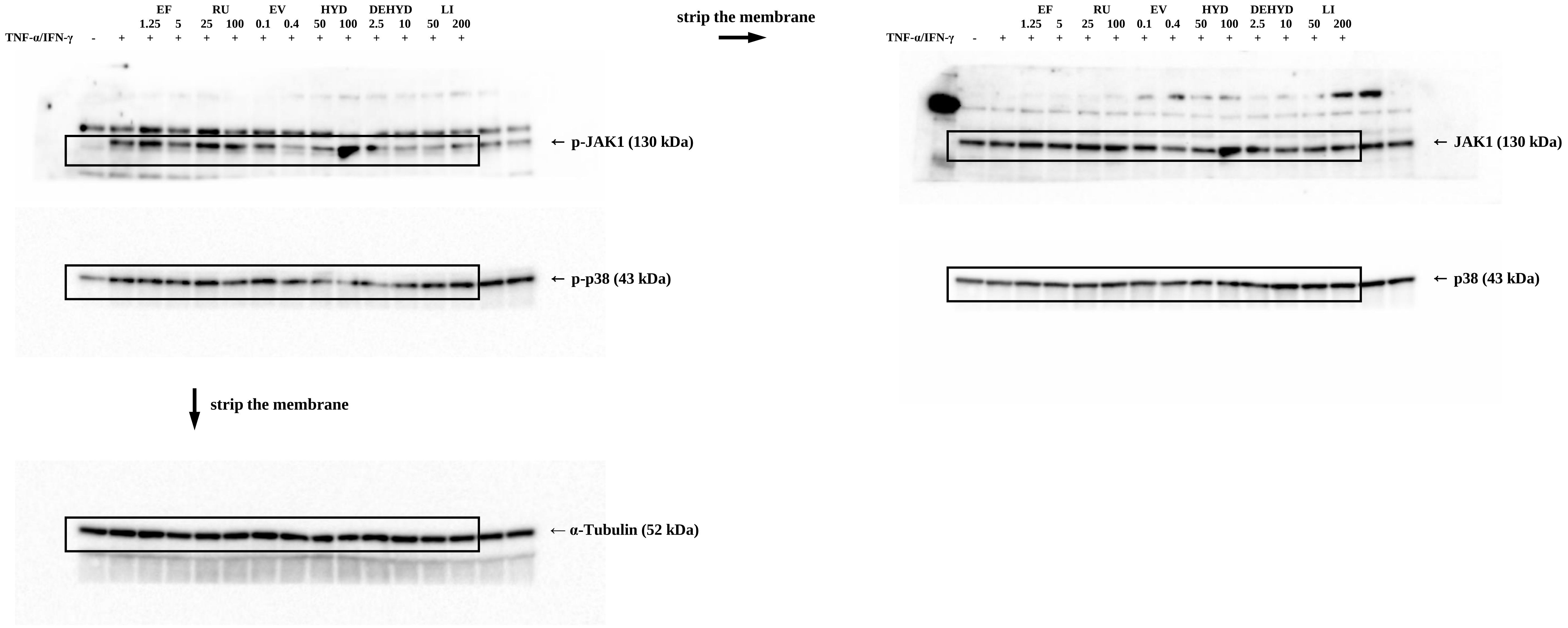

3rd Membrane #2

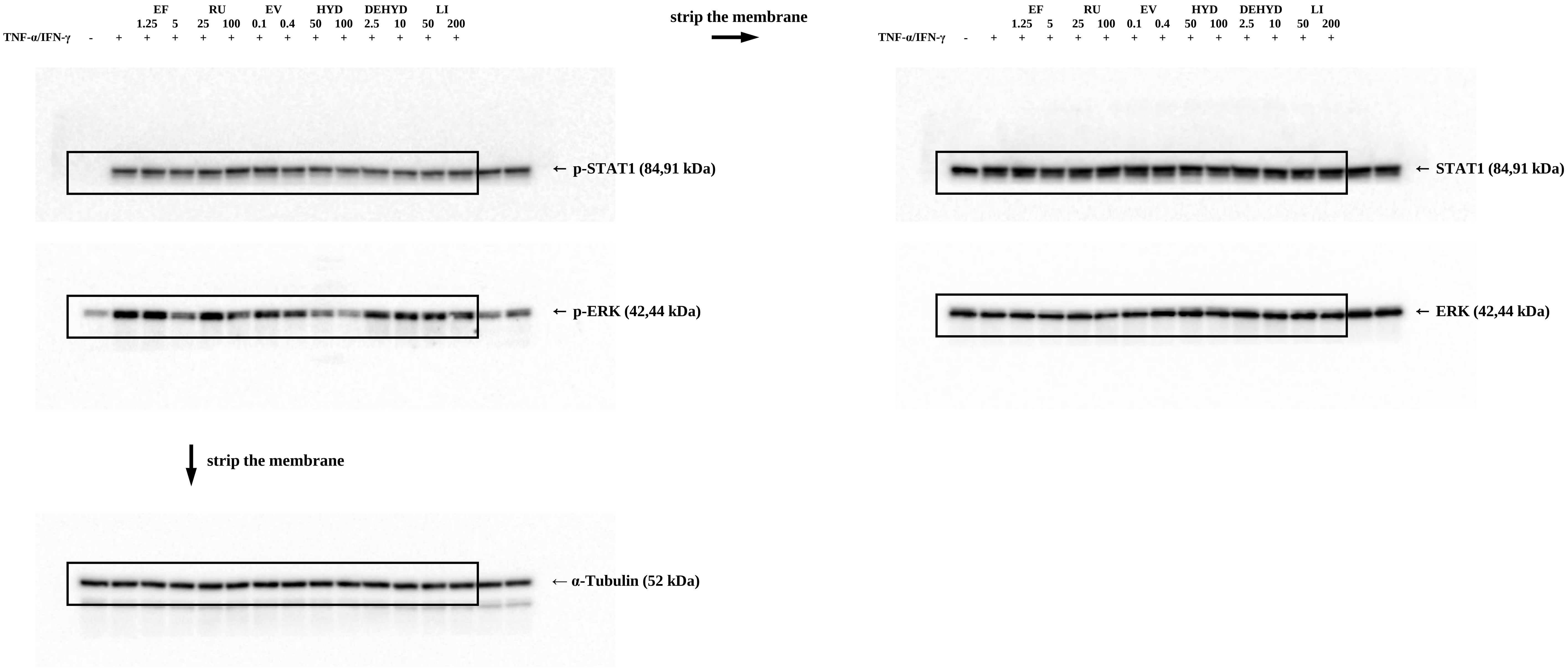

3rd Membrane #3

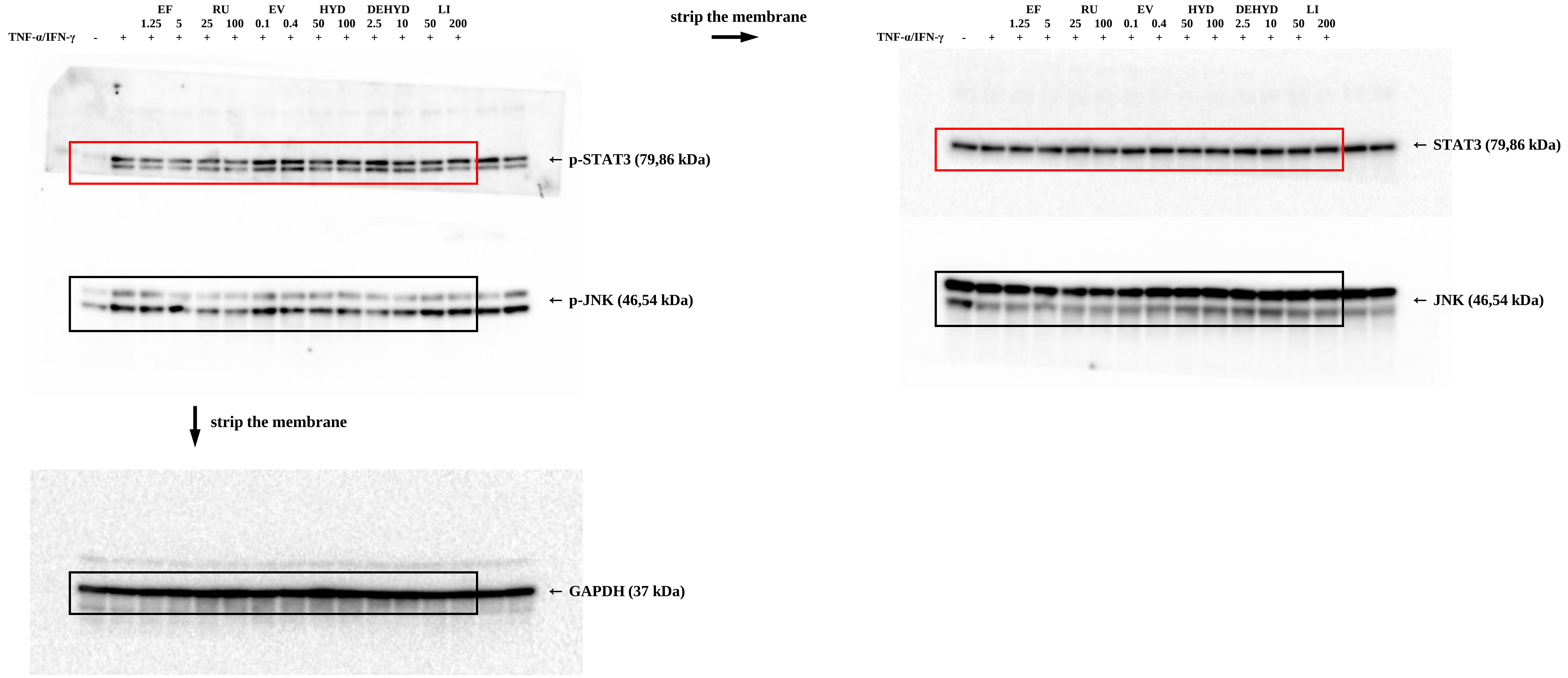

3rd Membrane #4

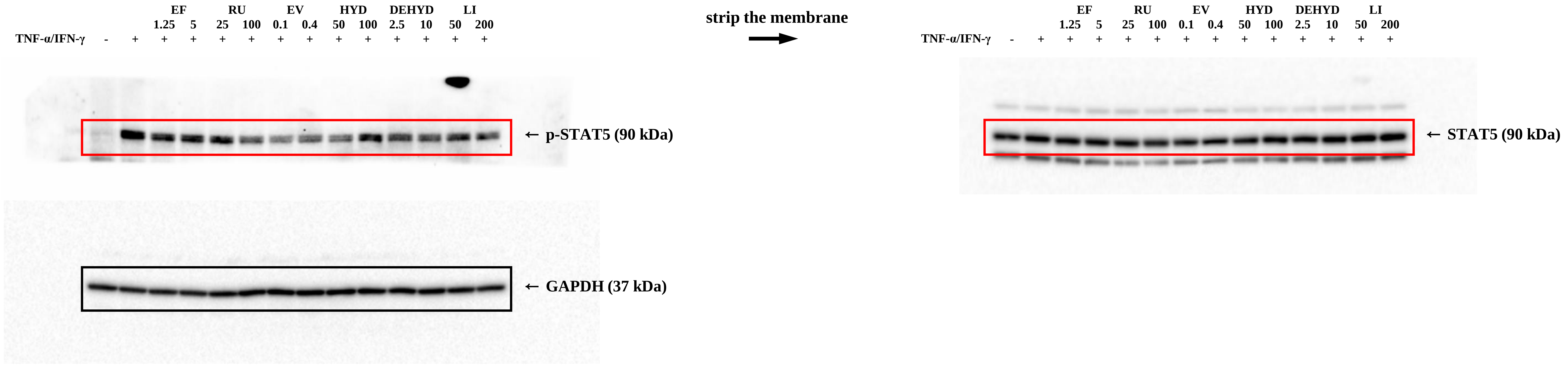

3rd Membrane #5

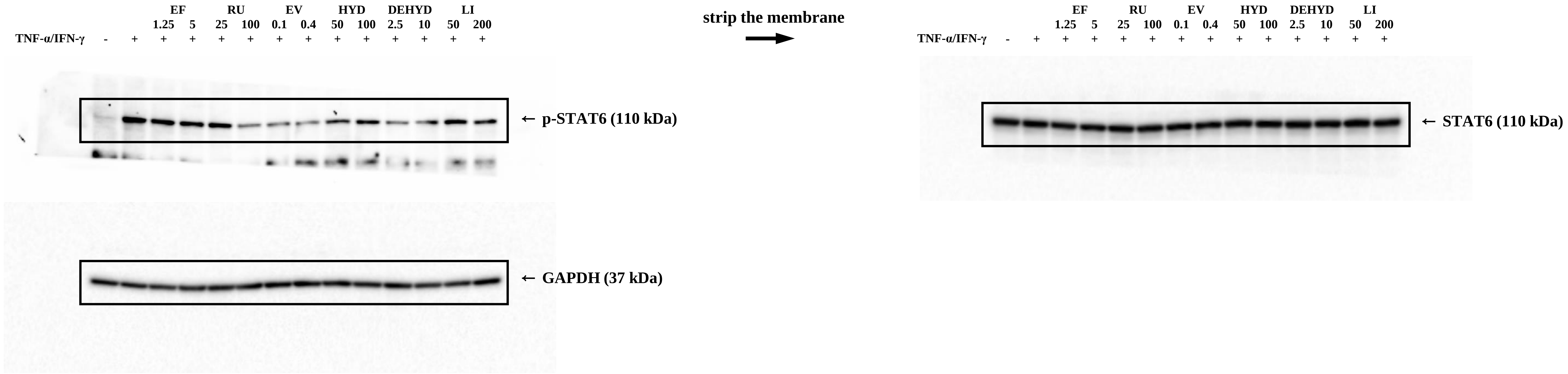

Supplement: Supplementary file 1 — Supplementary Figures. [file 41598_2023_50257_MOESM1_ESM.pdf]
