# Supplementary material for: Evodiae Fructus extract suppresses inflammatory response in HaCaT cells and improves house dust mite-induced atopic dermatitis in NC/Nga mice
Source: Sci Rep. 2024 Jan 4;14:472. doi: 10.1038/s41598-023-50257-3 (PMC10764943; doi:10.1038/s41598-023-50257-3)
Supplement: Supplementary file 2 — Supplementary Information 1. [file 41598_2023_50257_MOESM2_ESM.pdf]

Supplementary Information

Table S1. Information on cell lines and reagents

Category	Chemicals and reagents	Company
Cell line	HaCaT	CLS Cell Lines Service GmbH (Eppelheim, Baden-Württemberg, Germany)
Cell culture	DMEM<br>FBS<br>PBS<br>Penicillin and streptomycin	Thermo Fisher Scientific (Waltham, MA, USA)
Reagent	Acetonitrile (LC–MS grade)<br>Methanol (LC–MS grade)<br>Water (LC–MS grade)	Fisher Scientific Co., LLC (San Jose, CA, USA)
	Ammonium formate	Kanto Chemical Co., Inc. (Tokyo, Japan)
	Prednisolone<br>Silymarin	Sigma-Aldrich (St. Louis, MO, USA)
	Biostir-AD®	Biostir Inc. (Osaka, Japan)
	Entobar (pentobarbital sodium)	Hanlim Pharm. Co., Ltd. (Seoul, Republic of Korea)
	Formic acid	Fujifilm Wako Pure Chemical Co., Ltd. (Osaka, Japan)
	IFN-γ<br>TNF-α	R&D Systems Inc. (Minneapolis, MN, USA)
	10% Neutral-buffered formalin	BBC Biochemical (WA, USA)

Assay kit	CCK-8	Dojindo (Kumamoto, Japan)
	Corticosterone	MyBioSource, Inc. (San Diego, CA, USA)
	Histamine	Oxford Biomedical Research Inc. (MI, USA)
	IgE	Abcam (Cambridge, UK & Cambridge, Massachusetts, USA)
	MDC<br>RANTES<br>TARC	R&D Systems Inc. (Minneapolis, MN, USA)
Antibody	$CD4^+$ T cell<br>TSLP	Abcam (Cambridge, UK & Cambridge, Massachusetts, USA)
	ICAM-1<br>IL-4	Biorbyt Ltd. (Cowley Road, Cambridge, United Kingdom)
	Phospho- and total-JAK1<br>Phospho- and total-STAT1, 3, 5, 6<br>Phospho- and total-p38<br>Phospho- and total-ERK<br>Phospho- and total-JNK	Cell Signaling Technology, Inc. (Danvers, MA, USA)
	HRP-conjugated secondary antibodies	Jackson ImmunoResearch (West Grove, PA, USA)

Table S2. Compounds information

Code No.	Name	Molecular formula	CAS No.	Catalog No.	Purity (%)	Company
Comp.1	Rutaecarpine	$C_{18}H_{13}N_3O$	84-26-4	DR10962	99.0	Shanghai Sunny Biotech Co., Ltd. (Shanghai, China)
Comp.2	Evodiamine	$C_{19}H_{17}N_3O$	518-17-2	E3531	99.0	Merck KGaA (Darmstadt, Germany)
Comp.3	Evodol	$C_{26}H_{28}O_9$	22318-10-1	CFN98214	99.0	ChemFaces (Wuhan, China)
Comp.4	Dehydroevodiamine	$C_{19}H_{15}N_3O$	67909-49-3	BP0464	99.8	Chengdu Biopurify Phytochemicals Ltd. (Chengdu, China)
Comp.5	Limonin (Evodin)	$C_{26}H_{30}O_8$	1180-71-8	BP0866	99.1	Chengdu Biopurify Phytochemicals Ltd. (Chengdu, China)
Comp.6	Synephrine	$C_9H_{13}NO_2$	94-07-5	CFN99551	98.0	ChemFaces (Wuhan, China)
Comp.7	Evocarpine	$C_{23}H_{33}NO$	15266-38-3	TBP03451	99.7	Wuhan ChemNorm Biotech Co., Ltd. (Wuhan, China)
Comp.8	Dihydroevocarpine	$C_{23}H_{35}NO$	15266-35-0	TB03209	99.4	Wuhan ChemNorm Biotech Co., Ltd. (Wuhan, China)
Comp.9	Hydroxyevodiamine	$C_{19}H_{17}N_3O_2$	1238-43-3	CFN90347	98.2	ChemFaces (Wuhan, China)

Table S3. UPLC-MS/MS MRM analysis conditions for simultaneous quantification of compounds in EFE

UPLC conditions				MS conditions	
System	Acquity UPLC I-Class			System	Xevo TQ-XS
Column	Acquity UPLC BEH C18 column (2.1 mm × 100 mm, 1.7 μm)			Software	MassLynx v4.2
Column temp.	45°C			Ion source	ESI+ or ESI–
Sample temp.	5°C			Acquisition mode	MRM
Injection volume	2.0 μL			Capillary voltage	1.2 kV
Flow rate	0.3 mL/min			Cone gas flow	50 L/h
Mobile phase A	0.1% (v/v) aqueous formic acid with 5 mM ammonium formate			Desolvation gas flow	700 L/h
Mobile phase B	Acetonitrile			Desolvation temperature	500°C
	Time (min)	A (%)	B (%)	Source temperature	150°C
Gradient	Initial	80	20		
	0.1	80	20		
	14.0	5	95		
	15.0	0	100		
	15.1	80	20		
	18.0	80	20		

EFE, Evodiae Fructus 70% ethanol extract; ESI, electrospray ionization; MRM, multiple reaction monitoring; UPLC-MS/MS, ultra-performance liquid chromatography with tandem mass spectrometry.

Table S4. UPLC–MS/MS MRM conditions for simultaneous analysis of compounds in EFE

Code No.	Name	Ion mode	Molecular weight	MRM transition		Cone voltage (V)	Collision energy (eV)	Retention time (min)
				Precursor ion	Product ion			
Comp.1	Rutaecarpine	+	287.3	288.2	273.1	30	30	6.65
Comp.2	Evodiamine	+	303.4	304.3	134.1	30	20	6.37
Comp.3	Evodol	−	484.5	483.3	421.2	30	15	5.71
Comp.4	Dehydroevodiamine	+	301.3	302.3	286.2	30	37	2.88
Comp.5	Limonin	+	470.5	471.2	161.1	30	25	5.37
Comp.6	Synephrine	+	167.2	168.1	91.0	15	20	0.95
Comp.7	Evocarpine	+	339.5	340.4	186.1	30	35	10.92
Comp.8	Dihydroevocarpine	+	341.5	342.4	173.1	30	35	12.23
Comp.9	Hydroxyevodiamine	+	319.4	320.4	169.1	30	10	7.36

EFE, Evodiae Fructus 70% ethanol extract; MRM, multiple reaction monitoring; UPLC-MS/MS, ultra-performance liquid chromatography with tandem mass spectrometry.

Table S5. Parameters for simultaneous quantification of compounds in EFE using the UPLC–MS/MS MRM method

Code No.	Name	Linear range (μg/L)	Regression equation (y = ax + b)	r^2	LOD (mg/L)	LOQ (mg/L)
Comp.1	Rutaecarpine	10.00-1,000.00	y = 2,207.61x + 6,862.19	0.9974	0.16	0.48
Comp.2	Evodiamine	10.00-1,000.00	y = 19,680.50x + 106,932.00	0.9977	0.02	0.05
Comp.3	Evodol	10.00-1,000.00	y = 25.68x + 20.70	0.9955	3.05	9.15
Comp.4	Dehydroevodiamine	10.00-1,000.00	y = 94,682.80x - 788,577.00	0.9951	0.05	0.16
Comp.5	Limonin	5.00-500.00	y = 66.34x + 83.40	0.9982	1.65	4.96
Comp.6	Synephrine	5.00-500.00	y = 58.67x + 612.50	0.9985	0.95	2.85
Comp.7	Evocarpine	10.00-1,000.00	y = 77,956.00x - 437,727.00	0.9961	0.07	0.21
Comp.8	Dihydroevocarpine	5.00-500.00	y = 142,705.00x - 259,126.00	0.9981	0.02	0.05
Comp.9	Hydroxyevodiamine	10.00-1,000.00	y = 26.29x - 17.98	0.9970	1.42	4.25

EFE, Evodiae Fructus 70% ethanol extract; LOD, limit of detection; LOQ, limit of quantitation; MRM, multiple reaction monitoring; UPLC-MS/MS, ultra-performance liquid chromatography with tandem mass spectrometry; x, concentration (μg/L) of each reference compound; y, peak area of each reference compound.

Figure S1. Chemical structures of nine compounds in EFE

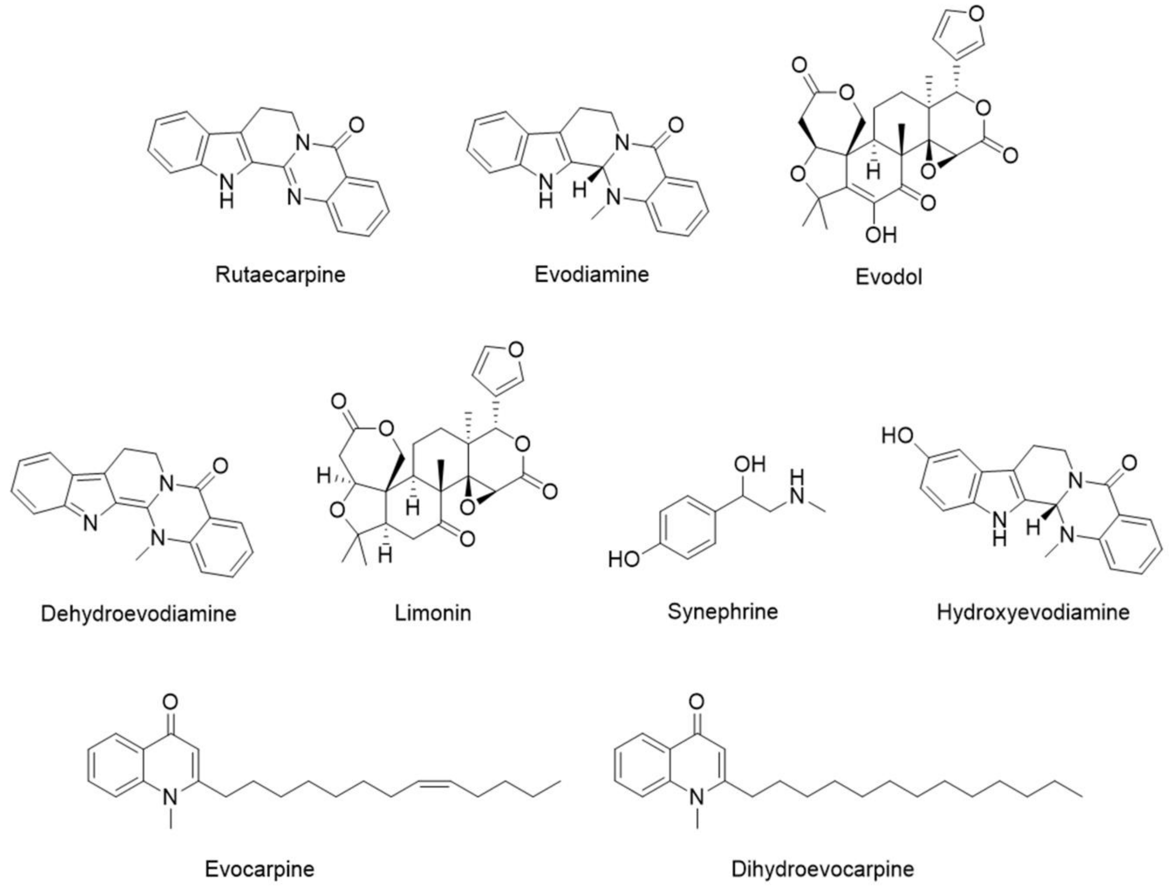

Figure S2. Cell viability assay procedure

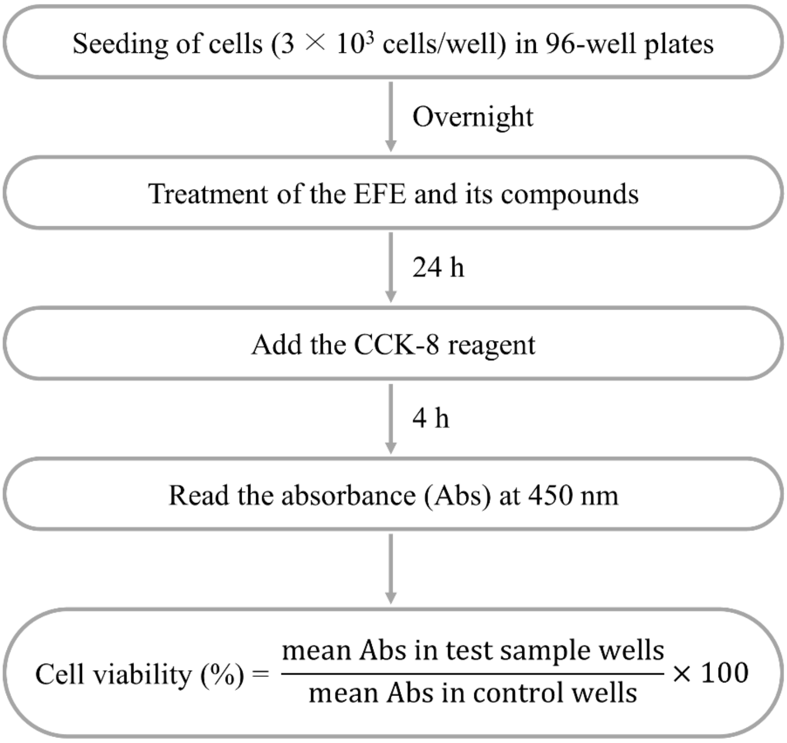

Figure S3. Schematic diagram of the experimental schedule

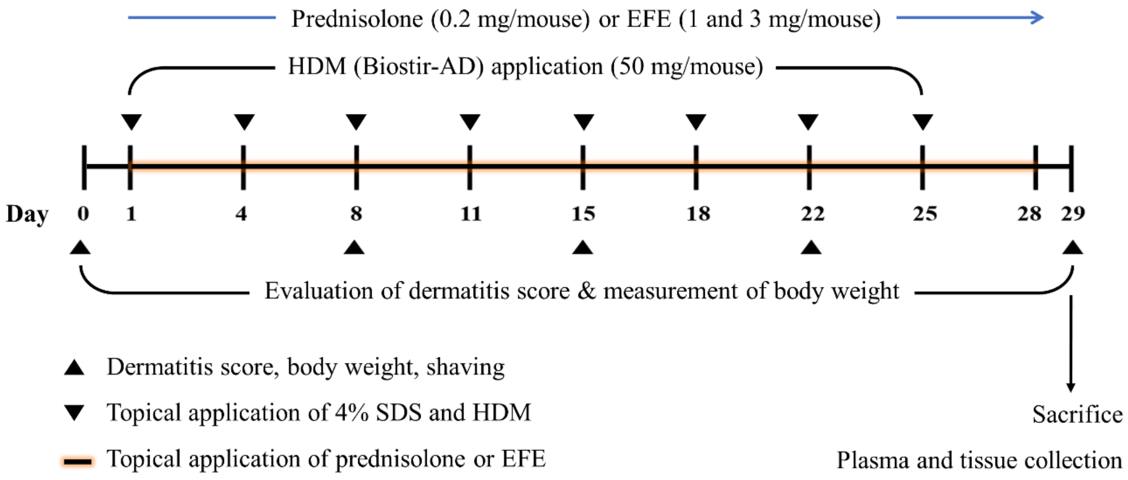

Mice were treated with 4% SDS (150 µL) and HDM (Biostir-AD ointment, 50 mg) to induce AD-like lesions twice a week for 4 weeks. Prednisolone (0.2 mg) and EFE (1 and 3 mg) were topical applied daily for 4 weeks. EFE, Evodiae Fructus 70% ethanol extract; HDM, house dust mite.

Figure S4. Cell viability of EFE, its compounds, and silymarin in HaCaT cells

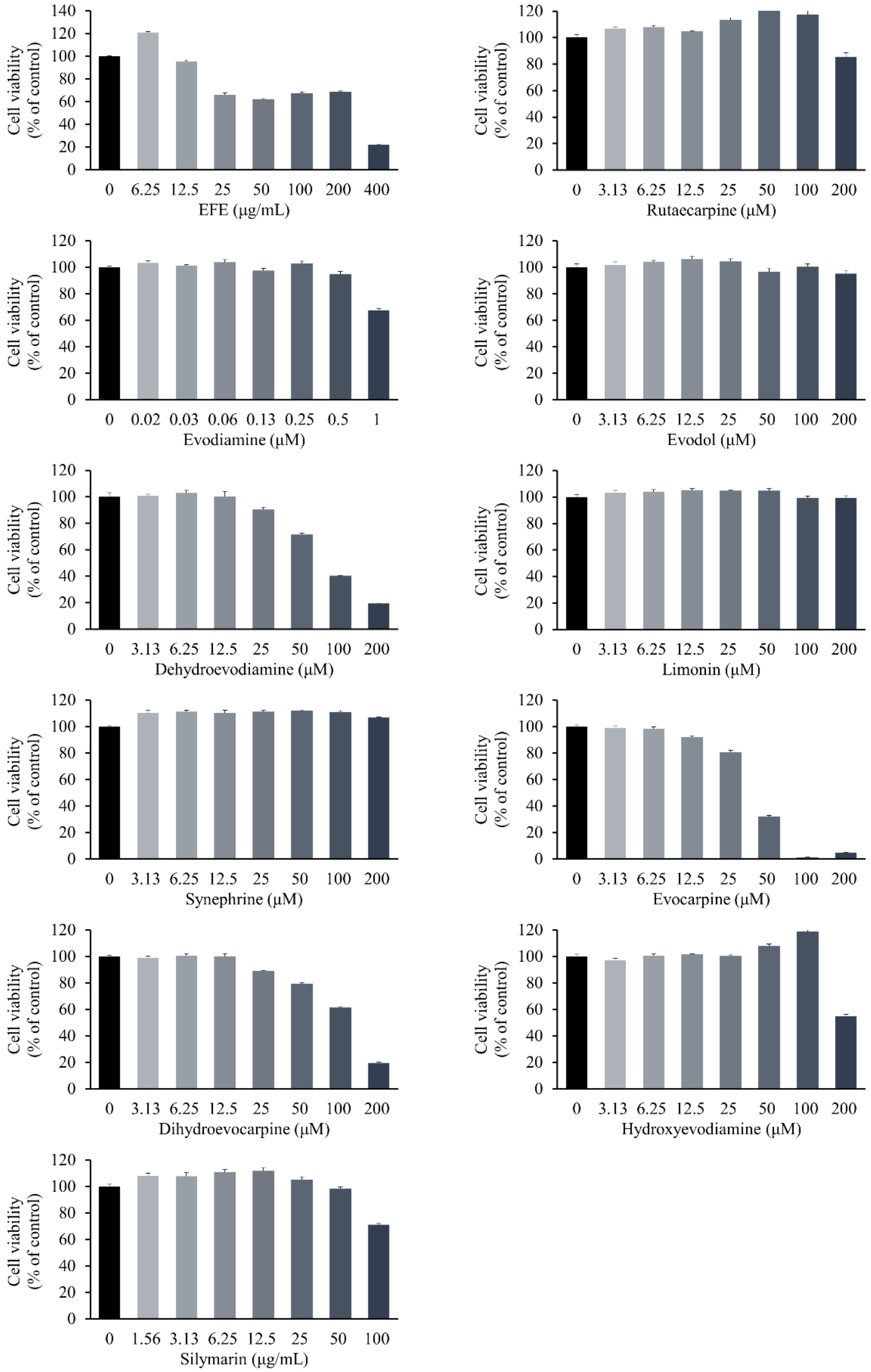

Data are expressed as mean ± SEM (n = 4).

Figure S5. Effect of EFE and its compounds on the production of chemokines in TI-stimulated HaCaT cells

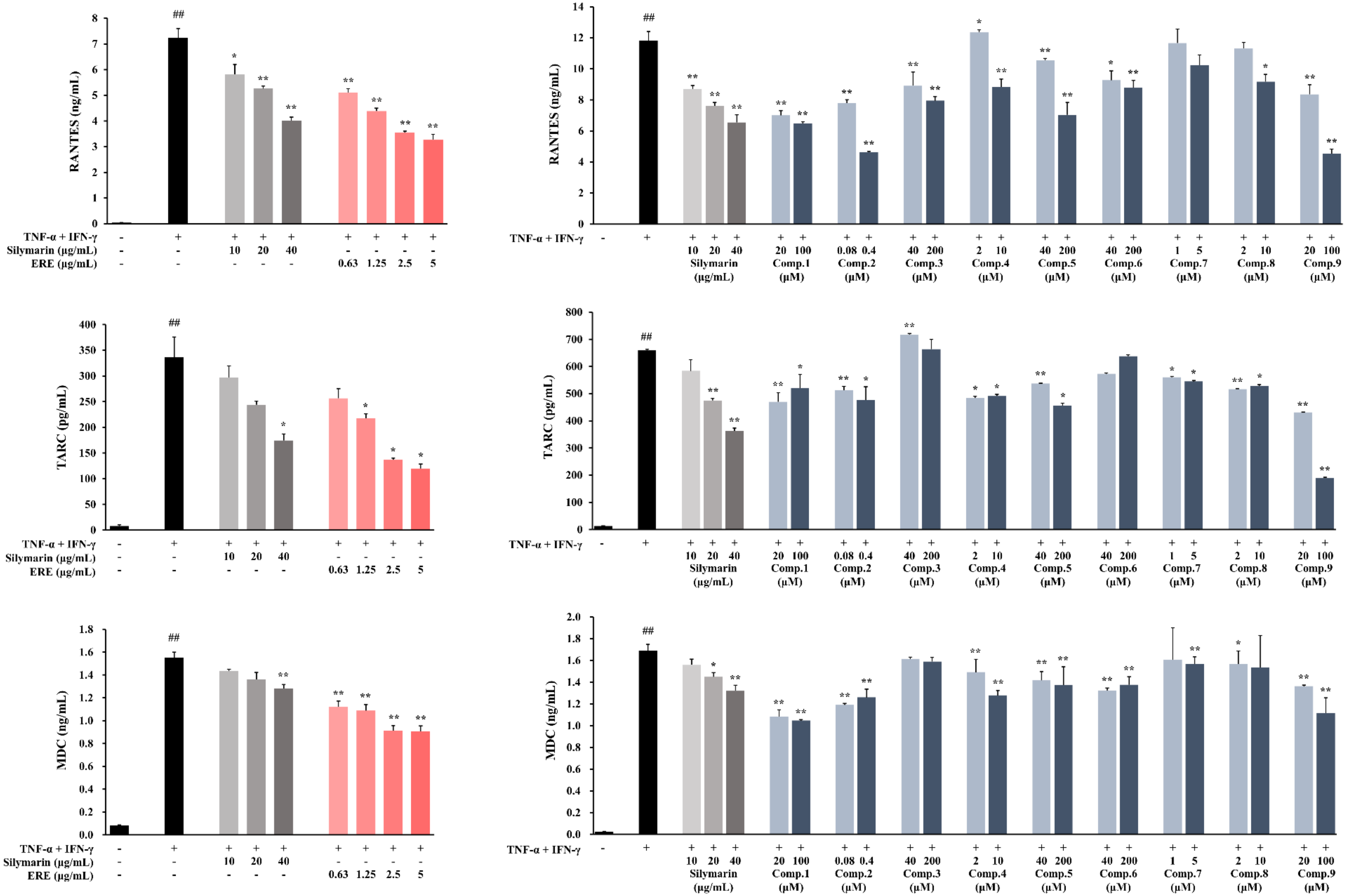

The cells were treated with EFE **(A)** and its compounds **(B)**, and stimulated with TI for 24 h. The levels of RANTES, TARC, and MDC in the supernatant were measured using ELISA kits. Silymarin was used as a positive control. Data are expressed as mean ± SEM (n = 3). $^{##}p < 0.01$ versus NC; $^{*}p < 0.05$ and $^{**}p < 0.01$ versus TI-stimulated cells. Comp.1, rutaecarpine; Comp.2, evodiamine; Comp.3, evodol; Comp.4, dehydroevodiamine; Comp.5, limonin; Comp.6, synephrine; Comp.7, evocarpine; Comp.8, dihydroevocarpine; Comp.9, hydroxyevodiamine; EFE, Evodiae Fructus 70% ethanol extract; NC, normal control; TI, TNF-α (10 ng/mL) and IFN-γ (10 ng/mL).
